# Microglial 5-LOX-activating protein antagonism alleviates leukotriene-driven neuroinflammation

**DOI:** 10.1007/s00401-026-03055-w

**Published:** 2026-08-04

**Authors:** Julia Konings, Fleur Mingneau, Serhii Chornyi, Cathrin E. Hansen, Rianne T. M. van der Burgt, Tessa G. van Elk, Laura Bolkaerts, Bo Batens, Gábor Tóth, Ingela Lanekoff, Frédéric M. Vaz, Alessio Cardilli, Marie Sels, Susanne M. A. van der Pol, Helga E. de Vries, Martin Giera, Oliver Werz, Sofie Kessels, Sanne G. S. Verberk, Jeroen F. J. Bogie, Merel Rijnsburger, Jerome J. A. Hendriks, Gijs Kooij

**Affiliations:** 1 Department of Molecular Cell Biology and Immunology, Amsterdam UMC Location Vrije Universiteit Amsterdam, De Boelelaan 1117, Amsterdam, The Netherlands; 2 Amsterdam Neuroscience, Amsterdam UMC, Amsterdam, The Netherlands; 3 MS Center Amsterdam, Amsterdam UMC Location VU Medical Center, Amsterdam, The Netherlands; 4 Department of Immunology and Infection, Biomedical Research Institute, Hasselt University, Diepenbeek, Belgium; 5 University MS Center, Pelt, Belgium; 6 Center for Proteomics and Metabolomics, Leiden University Medical Centre (LUMC), Leiden, The Netherlands; 7 Amsterdam Institute for Immunology & Infectious Diseases, Amsterdam, The Netherlands; 8 Department of Chemistry for Life Sciences, Uppsala University, Uppsala, Sweden; 9 Center for Excellence for the Chemical Mechanisms of Life, Uppsala University, Uppsala, Sweden; 10 Laboratory Genetic Metabolic Diseases, Amsterdam UMC, Amsterdam, the Netherlands; 11 Core Facility Metabolomics, Amsterdam UMC, Amsterdam, the Netherlands; 12 Department of Biochemistry, Cardiovascular Research Institute Maastricht, University of Maastricht, Maastricht, The Netherlands; 13 Department of Pharmaceutical/Medicinal Chemistry, Institute of Pharmacy, Friedrich Schiller University, Jena, Germany; 14 Jena Center for Soft Matter (JCSM), Friedrich Schiller University Jena, Jena, Germany; 15 Amsterdam Institute for Immunology and Infectious Diseases, Amsterdam UMC, Amsterdam, The Netherlands

**Keywords:** Multiple sclerosis, Leukotriene B_4_, 5-Lipoxygenase activating protein (FLAP), PA nano-DESI MSI,, Lipidomics, Neuroinflammation, Microglia, Experimental autoimmune encephalomyelitis (EAE)

## Abstract

In multiple sclerosis (MS), the chronic, unresolved nature of neuroinflammation within the central nervous system (CNS) remains a major obstacle for effective therapeutic intervention. This challenge arises primarily due to an incomplete understanding of the dysregulated inflammatory and pro-resolving pathways underlying MS lesion progression. Bioactive lipid mediators (LMs), biosynthesized through the coordinated actions of specific enzymes like lipoxygenases (LOX) and cyclooxygenases (COX), are key regulators of both the initiation and resolution of an inflammatory response; however, their spatial organization and functional role during MS pathology have not been fully elucidated. Here, by using pneumatically assisted nanospray desorption electrospray ionization (PA nano-DESI) mass spectrometry imaging and immunohistochemistry, we reveal an increase in the LM leukotriene B_4_ (LTB_4_) in human MS white matter compared to controls, with further enrichment in MS lesions relative to perilesional areas, alongside elevated microglial 5-LOX activating protein (FLAP) expression. Pharmacological antagonism of FLAP suppresses LTB_4_ biosynthesis in human-induced pluripotent stem cell (iPSC)-derived microglia with only marginal effects on the microglia transcriptional phenotype as determined by RNA sequencing. Moreover, in vivo FLAP antagonism ameliorates disease severity and spinal cord inflammatory gene expression in the experimental autoimmune encephalomyelitis (EAE) model, an animal model of MS, in both a prophylactic and therapeutic settings. This coincided with reduced local LTB_4_ biosynthesis and reduced levels of inflammatory monocytes within the spinal cord during EAE. Together these findings establish the FLAP/LTB_4_ axis as a driver of neuroinflammation and a druggable therapeutic target for chronic inflammatory CNS disorders like MS.

**Supplementary Information:**

The online version contains supplementary material available at https://doi.org/10.1007/s00401-026-03055-w.

## Background

Multiple sclerosis (MS) is a chronic inflammatory and demyelinating disease of the central nervous system (CNS). Lesions in the brain and spinal cord (SC) of people with MS (PwMS) across disease stages show various stages of demyelination as well as neuroinflammation, characterized by infiltration of peripheral immune cells and local (chronic) activation of tissue-resident microglia [10, 33]. On one hand, microglia contribute to demyelination and axonal degeneration through their chronic inflammatory activity; on the other hand, their roles in tissue repair and phagocytosis of myelin debris can promote resolution of inflammation [73], which can halt lesion progression and allow remyelination of the affected area [10, 33]. Current treatment options for PwMS are generally ineffective in fully preventing long-term disability progression and neurodegeneration across disease stages [22]. Despite the well-established link between inflammation, demyelination, and failed remyelination, the precise mediators driving these processes remain unclear. Hence, studying the balance between chronic inflammation and pro-resolution signals within the local metabolic environment represents a promising approach.

Lipid mediators (LM) are bioactive molecules that function as pivotal regulators of the initiation and resolution of inflammation [6, 61]. These metabolites are derived from omega-3 and -6 polyunsaturated fatty acids (PUFAs). PUFAs, such as arachidonic acid (AA, omega-6), can be released from generally membrane-bound lipids via cytoplasmic phospholipase A_2_, and can be subsequently oxygenated by various enzymes, such as cyclooxygenases (COX) and lipoxygenases (LOX), resulting in the biosynthesis of LMs, such as prostaglandins and leukotrienes (LTs) [47]. Interestingly, our recent findings indicate that the active enzymatic conversion of AA into bioactive LMs correlates with MS disability scores, as reflected by diminished AA and concomitant elevation of LMs in plasma, pointing to an upregulated local metabolic AA flux towards downstream effectors [6, 21].

One of these downstream metabolites is leukotriene B_4_ (LTB_4_). The first steps of LTB_4_ biosynthesis are catalyzed by the 5-LOX enzyme and facilitated by 5-LOX-activating protein (FLAP), which binds 5-LOX substrates and subsequently transfers them to the enzyme. The 5-LOX/FLAP complex is localized at the nuclear membrane and initiates the biosynthesis of LTA_4_, which can be further converted into LTB_4_ via epoxide hydrolysis [50, 74]. LTB_4_ has been recognized for its pivotal role in orchestrating and amplifying inflammatory responses through its role as a potent chemoattractant and activator of leukocytes [83]. Although the 5-LOX-LTB_4_ axis has been implicated in a well-established animal model for MS (experimental autoimmune encephalomyelitis, EAE) and other inflammatory diseases, the characterization of LTB_4_ and FLAP in the human brain under healthy or pathological conditions, and whether FLAP antagonism might offer therapeutic potential in the context of MS, remains largely unknown [31, 32, 41, 77, 82]. Targeted inhibition of FLAP may represent a more selective approach to regulate 5-LOX activity, constraining LT-driven inflammatory pathways while maintaining general 5-LOX function. This refined modulation may offer improved therapeutic efficacy and safety in neuroinflammatory conditions, including MS, compared to 5-LOX inhibition.

In the present study, we aimed to dissect the spatial and functional significance of FLAP and LTB_4_ in MS. By using mass spectrometry imaging (MSI), we found that LTB_4_ levels are increased within human MS WM lesions. Immunostainings demonstrated that the percentage of FLAP^+^ cells is concomitantly increased in MS lesion areas, predominantly in the microglial population. Accordingly, human-induced pluripotent stem cell (iPSC)-derived microglia showed increased FLAP expression under inflammatory conditions, and pharmacological targeting using a FLAP antagonist (FLAPa) resulted in reduced LTB_4_ production. Lastly, FLAPa treatment was applied in vivo to EAE mice, where it ameliorated disease scores both in prophylactic and therapeutic settings, accompanied by reduced LTB_4_ biosynthesis from its substrate AA as well as reduced levels of inflammatory monocytes within the spinal cord during EAE. Taken together, these findings identify the FLAP-LTB_4_ pathway as a key contributor to neuroinflammation in MS and support its candidacy as a novel therapeutic target to combat MS.

## Materials and methods

### Human tissue samples

Snap-frozen or formalin-fixed paraffin-embedded post-mortem (FFPE) human brain white matter (WM) tissue blocks were obtained from PwMS (total *n* = 16) and non-neurological controls (NNC) (total *n* = 12) and provided by the MS Center Amsterdam and the Netherlands Brain Bank. Relevant clinical information and experimental application are summarized in Table 1. All donors, or their next of kin, had given fully informed consent for autopsy and use of material for research purposes from the Netherlands Brain Bank under ethical approval by the Medical Ethics Committee of the University Medical Center in Amsterdam (2009/148), project number 1,127.

**Table 1:** Clinical and demographic data of MS and NNC subjects

Case ID	Age at death	Sex	PMD (h)	Cause of death	Type of MS	Tissue preservation	Lesion type	Applied techniques
NNC019	68	M	8:40	Euthanasia	n/a	FF, FFPE	n/a	MSI, IHC (TMEM119), mRNA
NNC095	68	M	7:35	Esophageal carcinoma, narrowing of coronary arteries	n/a	FFPE	n/a	IHC (5-LOX, TMEM119)
NNC215	79	M	9:00	Pneumonia and metastasized kidney carcinoma	n/a	FF	n/a	mRNA
NNC227	93	M	6:00	Cachexia, cerebrovascular accident/kidney failure	n/a	FF	n/a	mRNA
NNC254	75	F	5:40	Cachexia	n/a	FF	n/a	mRNA
NNC275	82	F	5:10	Pneumonia by hemothorax	n/a	FF	n/a	mRNA
NNC534	67	M	8:24	Cancer (bladder/bone)	n/a	FFPE	n/a	IHC (5-LOX)
NNC782	69	F	13:00	Pulmonary embolism	n/a	FFPE	n/a	IHC (5-LOX)
NNC880	77	M	11:25	Pneumonia	n/a	FFPE	n/a	IHC (TMEM119)
NNC945	59	F	8:10	Euthanasia, terminal COPD	n/a	FF	n/a	MSI, IHC (TMEM119, 5-LOX), mRNA
NNC989	71	F	7:50	Lung carcinoma	n/a	FF	n/a	MSI, IHC (TMEM119, 5-LOX), mRNA
NNC999	74	M	10:20	Euthanasia	n/a	FF	n/a	mRNA
MS16	77	F	9:45	Aspiration pneumonia	SPMS	FF, FFPE	A/I	MSI, IHC (TMEM119, 5-LOX), mRNA
MS31	53	F	5:50	Euthanasia	PMS	FFPE	A/I	IHC (TMEM119, 5-LOX)
MS32	54	M	unk	Progressive dyspnea	unk	FF	A (type I*)	MSI, mRNA
MS42	82	F	7:30	Cardiac arrest, sudden death	PPMS	FF	A/I	MSI, mRNA
MS46	51	F	9:10	Euthanasia	SPMS	FF, FFPE	A	MSI, IHC (TMEM119, 5-LOX), mRNA
MS51	47	M	7:15	Urosepsis with organ failure	SPMS	FF	unk	mRNA
MS60	60	F	5:05	Euthanasia	SPMS	FF	A/I	MSI, mRNA
MS68	48	F	9:20	Pneumonia	unk	FF	A/I	MSI, mRNA
MS71	67	M	7:55	Euthanasia	unk	FFPE	A/I	IHC (TMEM119)
MS85	67	F	11:25	Pneumonia	unk	FFPE	A/I	IHC (TMEM119, 5-LOX)
MS100	71	F	7:05	Cachexia with PMS and metastatic breast cancer	PMS	FF	A/I	mRNA
MS115	56	M	6:15	Respiratory insufficiency due to MS, assumed pneumonia	PPMS	FFPE	A/I	IHC (5-LOX)
MS116	66	F	9:30	Euthanasia	SPMS	FF	CIA (type I*)	MSI, mRNA
MS120	73	M	8:45	Urosepsis	SPMS	FF	A/I	mRNA
MS139	56	M	8:00	Pneumonia	SPMS	FF	unk	mRNA
MS298	53	F	10:45	Euthanasia	PMS?	FF	unk	mRNA

*PMD* post-mortem delay, *unk* unknown, *n/a* not applicable, *mRNA* messenger RNA, *PMS* progressive MS, *PPMS* primary progressive MS, *SPMS* secondary progressive MS, *FF* freshly frozen, *FFPE* formalin-fixed paraffin-embedded, *CIA* chronic inactive, *A/I* mixed active/inactive, *A* active, *MSI* mass spectrometry imaging

^*^Only white matter part of lesions was used

### Mice

Female C57BL/6 J mice were purchased from Envigo. Animals were maintained on a 12 h light/dark cycle with free access to water and a standard chow diet. All experiments were conducted in accordance with the institutional guidelines and approved by the Ethical Committee for Animal Experiments of Hasselt University (ID202426).

### Experimental autoimmune encephalomyelitis

Mice (11 weeks old) were subcutaneously immunized with 200 µg myelin oligodendrocyte glycoprotein peptide (MOG_35-55_) emulsified in 200 µl complete Freund’s adjuvant with *Mycobacterium tuberculosis* according to the manufacturer’s guidelines (EK-21110 kit; Hooke Laboratories). Immediately after immunization and after 24 h, mice received an intraperitoneal (i.p.) injection of 100 ng pertussis toxin (EK-2110 kit; Hooke Laboratories) to induce EAE. Starting 9 days (prophylactic setup) or 17 days (therapeutic setup) post-immunization, mice were treated daily with a FLAP antagonist (fiboflapon, 2 mg/kg, i.p., daily) or vehicle (100 µL PBS, i.p., daily). Mice were weighed daily and evaluated for neurological signs of the disease in a blinded fashion according to the manufacturer’s mouse EAE scoring guide (0: no clinical symptoms; 1: tail paralysis; 2: tail paralysis and partial hind limb paralysis; 3: complete hind limb paralysis; 4: paralysis to the diaphragm; 5: death by EAE). At the end of the experiment, mice were sacrificed with a lethal dose of dolethal (200 mg/kg, i.p.) and perfused with PBS with heparin (20 U/ml). Afterwards, SC were collected, snap-frozen, and stored at −70 ºC for lipidomics, immunostainings, and qRT-PCR.

### Human hiPSC-derived microglia

Human-induced pluripotent stem cells (hiPSCs) were generated from one NNC with the approval of the LUMC scientific ethical committee and obtained informed consent (NL45478.058.13/P13.080) [7]. hiPSC-derived microglia were generated according to the protocol of Kenkhuis et al. and Haenseler et al. [20, 30]. Briefly, mesodermal embryoid bodies were developed from hiPSCs, which were plated and supplemented with medium containing macrophage colony-stimulating factor (rhM-CSF, 100 ng/ml; 300–25, PeproTech) and Interleukin 3 (IL-3, 25 ng/ml; #200–03-B, PeproTech). Precursors were differentiated into hiPSC-derived microglia in 2 weeks using IL-34 (100 ng/ml; #200–34, PeproTech) and granulocyte–macrophage (GM)-CSF (10 ng/ml; #300–03, PeproTech) while changing the medium every other day. Matured hiPSC-derived microglia were left untreated (resting), or were subjected to either treatment with IL-4 (20 ng/ml; #11,340,043, ImmunoTools) and IL-13 (20 ng/ml; #200–13, PeproTech) for 48 h (pro-resolution) or with lipopolysaccharide (LPS) (100 ng/ml; L2630, Sigma-Aldrich) and Interferon gamma (IFNγ, 20 ng/ml; #300–02, PeproTech) (pro-inflammatory) for 24 h. As quality control, phenotypes were assessed for every batch by qPCR analysis. For lipidomics experiments, cells were washed twice with PBS and pretreated with a selective FLAP antagonist (fiboflapon 0.5 μM; HY-15874, MedChemExpress) or vehicle (0.01% DMSO, Sigma-Aldrich) in PBS supplemented with 0.1% glucose and 1 mM CaCl_2_ for 15 min, followed by a 15 min treatment with the calcium ionophore A23187 (2.5 μM; C7522, Sigma-Aldrich) added directly to the medium to stimulate LM production. For remaining experiments, cells were washed twice with PBS and treated with FLAP antagonist (fiboflapon 0.5 μM; HY-15874, MedChemExpress) or vehicle (0.01% DMSO, Sigma-Aldrich) in culturing medium for 24 h.

### Mass spectrometry imaging

Snap-frozen human tissue blocks were cryo-sectioned (CryoStar NX70, Thermo Fisher Scientific) into 10 µm sections, after which they were thaw-mounted onto super-frost microscopy glass slides and stored at −70 °C until further use. For mass spectrometry imaging (MSI), tissue slides were thawed and washed by submerging them in Milli-Q water (18.2 MΩ) six times for 1 min, in order to prevent the interference of excess salt in the analysis. Subsequently, tissues were dried under N2 flow at room temperature (RT). To visualize tissue LTB_4_ distribution, pneumatically assisted nanospray desorption electrospray ionization mass spectrometry imaging (PA nano-DESI MSI) was performed as described previously [13, 21]. To summarize, the PA nano-DESI probe consisted of two fused silica capillaries (50 µm inner diameter, 150 µm outer diameter) placed at a ca. 90° angle relative to one another. Extraction solvent was delivered through the primary capillary with a syringe pump (KD Scientific) at a flow rate of 0.5 µL/min, forming a liquid bridge at the intersection of the primary and secondary capillary. The extraction solvent was composed of acetonitrile:methanol v/v 9:1 (0.1% formic acid) spiked with 10 ppm ^107^Ag^+^ for adduct formation and with 0.5 µM LTB_4_-d_4_ internal standard (Cayman Chemical) for normalization and quantitation [14]. Co-axial nitrogen flow was applied to the tip with a gas pressure of 3.5 bar to provide pneumatic assistance.

Slides were directed under the probe using an XYZ stage (Zaber Technologies) controlled by an in-house developed LabView program [35]. Lines were acquired scanning at 50 µm/s in the X direction and stepping 150 µm in the Y direction, resulting in pixels of ~ 25 × 150 µm. An IQ-X mass spectrometer (Thermo Fisher Scientific) was used, with a capillary voltage of 3.5 kV and a heated capillary temperature of 275 °C. Data were acquired by alternating full scan (*m/z* 200–1100) and targeted Selected Ion Monitoring (tSIM) (*m/z* 425–475) events at a resolution of 120 000 (at *m/z* 200), and the orbitrap automatic gain control target was 50% and 40%, respectively. Ion images were created with the in-house developed ion-to-image (i2i) application [40] using 5 ppm mass tolerance, and an automatic contrast adjustment was applied to the 99th percentile of intensities. Endogenous intensities were normalized pixel-by-pixel to the internal standard in i2i to generate quantitative ion images.

#### Region selection and quantification

In order to pathologically characterize the NNC and MS tissue, consecutive brain slices of the tissues applied to PA nano-DESI MSI were stained for myelin proteolipid protein (PLP) and Human Leukocyte Antigen-DR isotype (HLA-DR) and scanned at 20 × magnification with the Olympus VS200 slide scanner (Evident). Using QuPath software (version 0.4.4) [4], the total WM tissue area was manually outlined for all cases (excluding grey matter), after which representative brain areas based on pathological classification were selected as regions of interest (ROIs) for paired analysis on the MS cases. In short, ROIs were based on PLP and HLA-DR immunoreactivity to assess myelination and neuroinflammation, respectively, following established histopathological criteria for the classification of MS lesions [33]. This resulted in three ROIs per MS tissue corresponding to the demyelinated lesion area, lesion rim, and peri-lesion (distant from the lesion). Total WM tissue outlines and ROIs were transferred into the i2i application [40], and quantities were extracted using one-point calibration defined in µM/pixel unit. Statistical analysis was performed with GraphPad Prism version 10.2.

### Immunostainings

For immunostainings, formalin-fixed paraffin-embedded human WM tissue blocks were sectioned at 5 μm, deparaffinized and washed with MilliQ (Millipore), followed by heat-mediated antigen retrieval for 10 min (10 mM sodium citrate buffer, pH 6), after which sections were brought to RT on ice for 30 min. Frozen human WM tissue blocks and mouse SC were sliced in, respectively, 5 μm and 10 μm sections (in longitudinal orientation for SC), dried and fixed with 4% PFA for 10 min at RT. All sections were washed with PBS and blocked with PBS containing 10% normal species serum (NSS) and 0.05% Tween 20 (Sigma-Aldrich) for 30 min. After that, primary antibodies for HLA-DR/MHCII (1:500; Hybridoma), PLP (1:300; Serotec, MCA839G), 5-LOX (1:150; Novus Biologicals, NBP2-46,513), FLAP (1:100, Novus Biologicals, NBP1-84,666), IBA1 (1:500; Abcam, ab5076), TMEM119 (1:50; R&D Systems, MAB10313), CD3 (1:200; Agilent Technologies, A0452), and CD45 (1:100; BD Biosciences, 553,076) diluted in PBS containing 1% NSS and 0.05% Tween20 were applied and sections were incubated overnight at 4 °C in the dark. Then sections were washed with PBS and incubated for 1 h at RT in the dark with the Alexa fluorophore-conjugated secondary antibodies diluted in PBS containing 0.05% Tween 20. Sections were washed with PBS, after which Hoechst (33,258, Thermo Fisher Scientific) was diluted in PBS to a final concentration of 10 μg/mL and applied for 1 min in the dark for nuclear staining and washed again with PBS. Finally, sections were mounted with Mowiol and a coverslip (Menzel-Glaser, thickness #1.5), and stored at 4 °C in the dark until image acquisition.

### Microscopy and image acquisition

Neuropathological characterization images for PLP and HLA-DR immunoreactivity from the MSI cohort were taken at the Olympus VS200 slide scanner using a 20 × overview scan and 60 × images (Olympus X line, 1.42 NA, oil) within the respective ROIs used previously for lipid quantification [21]. Images were deconvolved using Huygens Professional 21.10 software (scientific volume imaging B.V.). For all other immunostainings in human tissue, slides were imaged using the Akoya Vectra Polaris Automated Quantitative Pathology Imaging System, first taking a 10 × overview scan, after which ROIs were imaged with a 40 × air objective. Spectral unmixing using inForm (version 3.0, Akoya Biosciences) was applied to ROI images, which were then batch-analyzed using NIS elements (version 5.30.03, Nikon Europe B.V). Positive cells were defined by the overlap of nuclear and marker of interest staining based on thresholding, extracting an output of cell counts and mean fluorescent intensity within cell (marker) masks. Mouse SC were imaged in their entirety at the Olympus VS200 slide scanner using a 20 × overview scan. Images were analyzed in QuPath version 0.5.1 [4], using cell detection based on thresholding and positive cell classification resulting from trained machine learning classifiers per channel, followed by batch analysis.

### RNA isolation and real-time quantitative polymerase chain reaction (qRT-PCR)

Treated hiPSC-derived microglia were washed with PBS, followed by RNA extraction using TRIzol (#15,596–018, Thermo Fisher Scientific). TRIzol extraction was applied to mouse SC for the FLAPa EAE as well. For remaining qPCRs in mouse SC as well as for human brain tissue blocks, RNA was extracted using the Rneasy Lipid Tissue Mini Kit (#174,804, Qiagen). RNA quantity was measured using Nanophotometer (Implen, Westlake Village USA). After that, cDNA was synthesized using the High-Capacity cDNA Reverse Transcription Kit (#4,368,813, Thermo Fisher Scientific). Transcripts of interest were visualized and measured using SYBR Green (#4,309,155, Thermo Fisher Scientific) in the QuantStudio^TM^3 Real-Time PCR system (#A28567, Thermo Fisher Scientific). The ${2}^{-\Delta \Delta }$ CT relative quantification method was applied to normalize the expression to the housekeeping gene. For hiPSC-derived microglia, this was *POLRF2*, and reported measurements are the result of technical triplicates from five independent experiments. In the case of tissue application, each measurement represented one donor, with *ACTB* as a housekeeping gene for human brain samples and a combination of either *Actb*, *Hprt* and *Gapdh* (FLAPa EAE) or *Cyca* and *Tbp* (remaining qPCRs) for mouse SC samples. Primer sequences are summarized in Table 2.

**Table 2:** Primer details

Target	Forward primer (5'-3')	Reverse primer (5'-3')
Human		
* ALOX5*	AAG TAC ATC ACG CTG AAG ACG	CGG TGT TGC TTG AGA ATG TG
* ALOX5AP*	TCA TCA GCG TGG TCC AGA AT	CAC AGT TCT GGT TGG CAG TGT
* POLR2F*	GAA CTC AAG GCC CGA AAG	TGA TGA TGA GCT CGT CCA C
* ACTB*	GGG AAA TCG TGC GTG ACA TTA AG	TGT GTT GGC GTA AGG TCT TTG
Mouse		
* Alox5*	TCT TCC TGG CAC GAC TTT GCT G	GCA GCC ATT CAG GAA CTG GTAG
* Alox5ap*	TGC AGT CCA GAG TAC CAC AAG G	GTT CTT TGC CCA CAA GGT GGA G
* Ccl2*	GCC TCC ATC TTC ACG ACA GTG T	GTG AGG TTT GCC AAA GGC ACA C
* Ccl4*	GAA ACA GCA GGA AGT GGG AG	CAT GAA GCT CTG CGT GTC TG
* Ccl5*	GGA GTA TTT CTA CAC CAG CAG CAA	GCG GTTC CTT CGA GTG ACA
* Il1b*	TGG ATG CTC TCA TCA GCA CAG	GAA ATG CCA CCT TTT GAC AGT
* Il6*	TGT CTA TAC CAC TTC ACA AGT CGG AG	GCA CAA CTC TTT TCT CAT TTC CAC
* Nos2*	AAA ACC CCT TGT GCT GTT CTC	ATA CTG TGG ACG GGT CGA TG
* Tnfa*	CCA GAC CCT CAC ACT CAG	CAC TTG GTG GTT TGC TAC GAC
* Cyca*	GCG TCT CCT TCG AGC TGT T	AAG TCA CCA CCC TGG CA
* Tbp*	ATG GTG TGC ACA GGA GCC AAG	TCA TAG CTA CTG AAC TGC TG
* Actb*	TAT AAA ACC CGG CGG CGC A	CAT CCA TGG CGA ACT GGT GG
* Hprt*	TCC TCC TCA GAC CGC TTT TTG	TCA TCG CTA ATC ACG ACG CTG
* Gapdh*	GAC AAC TCA TCA AGA TTG TCA GCA	TTC ATG AGC CCT TCC ACA ATG

### RNA isolation and Quant Seq 3’mRNA-seq

For RNA sequencing applications, hiPSC-derived microglia were washed with PBS and lysed in 275 µl Buffer RLT Plus (#1,053,393; Qiagen), after which they were scraped, collected, and stored at −70 °C until further use. Cell lysates were thawed on ice, after which RNA was extracted using the RNeasy Plus Micro kit (#74,034; Qiagen). Obtained RNA concentrations were measured using the Qubit® 3.0 Fluorometer (ThermoFisher), and the Quant Seq 3’ mRNA-Seq V2 Library Prep Kit FWD with Unique Dual Indices (#UDI12A_0001-0096; Lexogen) was used according to the manufacturer’s instructions to prepare the sequencing libraries. Quality control was assessed using the 4200 TapeStation (Agilent Technologies). Sequencing libraries were pooled equimolarly, and sequencing was performed on a NextSeq 2000 (Illumina). Sequencing data were pre-processed by Lexogen Bioinformatics Services (Vienna, Austria) using their in-house pipeline, which includes quality control and adaptor trimming, alignment to the most recent human reference genome (homo_sapiens_GRCh38_ensembl_release_10 7_ERCC_SIR), and PCR deduplication through unique molecule identifier (UMI) processing.

#### Differential expression analysis

Downstream bioinformatics was applied in R (v4.3.2) using RStudio (v2023.12.1) [68, 69]. Data processing and normalization and subsequent differential expression (DE) analysis were executed using the edgeR (v4.0.16) and limma (v3.58.1) packages [8, 53]. Principal component analysis (PCA) plots visualizing sample clustering across conditions were generated using ggplot2 (v3.5.1) [78]. Differentially expressed genes (DEGs) were based on an adjusted *p* value of less than 0.05 and an absolute log_2_FC greater than 1, which were depicted in volcano plots produced using the EnhancedVolcano (v1.20.0) package [5]. Gene set enrichment analysis (GSEA) based on the ranked log_2_FC gene list was used to identify enriched gene ontology (GO) terms, using the clusterProfiler (v.4.4.4) package [79].

### Lipidomics and compound identification

After applying in vitro treatment, hiPSC-derived microglia were lysed with 1500 µL of methanol (300,000 cells/well in a 12-well plate with 500 µL of supernatant), cells were scraped, and the suspensions were kept at −70 °C. SC samples (50 mg of wet weight) were homogenized in 200 µL water with stainless steel beads (0.9 – 2.0 mm) using a Next Advance Bullet Blender (precooled, 5 min at speed 10). All samples were supplemented with 4 µL of an internal standard solution consisting of deuterated lipids LTB_4_-d_4_ (50 ng/mL), ( ±) 15-hydroxyeicosatetraenoic acid-d_8_ (50 ng/mL), prostaglandin E2-d_4_ (50 ng/mL), 8-iso prostaglandin F_2α_-d_4_ (100 ng/mL), docosahexaenoic acid–d_5_ (500 ng/mL) and 14(15)-epoxy-eicosatrienoic acid-d_11_ (50 ng/mL) (Cayman Chemical). SC homogenates were supplemented with 600 µL of methanol and equilibrated at −20 °C.

The samples were centrifuged for 10 min (16,200 g, 4 °C) and the supernatants were diluted with H_2_O to achieve a final methanol concentration of 21% (*v/v*), and the pH was adjusted to 3.5 using formic acid (VWR, 84,865.180). Lipids were extracted by solid-phase extraction (SPE) using C18 cartridges (Sep-Pak Vac3, 3 cc, 200 mg). Cartridges were pre-equilibrated with methanol (LC–MS grade; Merck) and water (LC–MS grade; VWR) before sample loading. After washing with water and n-hexane (Sigma-Aldrich), lipids were eluted with methyl formate (spectrophotometric grade; Sigma-Aldrich). Eluates were dried at 40 °C under a gentle nitrogen stream and reconstituted in 100 µl MeOH:H_2_O (40% *v/v*). Samples were analyzed using a targeted LC–MS/MS method as described previously [17]. Chromatographic separation was performed on a high-performance liquid chromatography (HPLC) system equipped with a C18 column (Kinetex, 00B-4475-AN; Phenomenex) and a C8 precolumn (Kinetex, AJ0-8784; Phenomenex) maintained at 50 °C. Lipids were separated using a linear gradient between solvent A (water + 0.01% [*v/v*] acetic acid) at a flow rate of 0.4 mL/min. The gradient profile was as follows: 0–1 min, 70% A; 1–1.1 min, 70–55% A; 1.1–2 min, 55–46.5% A; 2–4 min, 46.5–44.5% A; 4–7 min, 44.5–10% A; 7–7.1 min, 10–0% A; 7.1–9.5 min, 0% A; 9.5–10.5 min, 0–70% A (equilibration). Detection was performed on a QTrap 6500 tandem mass spectrometer (ScieX) operated in negative electrospray ionization mode. Individual lipids were identified based on precursor ion *m/z* (Q1), characteristic product ions *m/z* (Q3), and retention time. The area ratio to the corresponding internal standard was converted to absolute amounts (ng) using an external calibration curve. For SC samples, the obtained amounts were normalized for tissue weight. The amount of LTB_4_ is reported as a sum of three detected stereoisomers that separate based on retention time: LTB_4_, 6-trans-LTB_4_ and 6-trans-12-epi-LTB_4_. For the in vivo experiments, the ratio of this sum to arachidonic acid was calculated (reported as LTB_4_:AA).

For compound identification of fiboflapon in the CNS, EAE mice brains (5 mg per sample) were homogenized using steel beads, 1.5 ml of chloroform/methanol were added. The mixtures were sonicated in a water bath at room temperature for 10 min, followed by centrifugation at 16,000 g for 10 min. The liquid phase was transferred into glass vials and evaporated under a stream of nitrogen at 50 °C. The residue was dissolved in 60 μl of chloroform/methanol (1:1, v/v), and 10 μl of the solution was injected into the HPLC–MS system. Reversed-phase HPLC was used to separate components of the samples; for this, a C_18_ Acquity HSS T3 column was used. A Q Exactive Plus Orbitrap mass spectrometer was used in positive electrospray ionization mode. Fiboflapon was identified in brain samples as [C_38_H_43_N_3_O_4_S + H]⁺ peak (predicted m/z = 638.30527) that eluted at 2 min.

### Flow cytometry

At the disease peak (17 days post-immunization), whole blood, spleen, and SC were isolated from EAE mice. A single-cell suspension was generated from spleen by mechanical transfer through a 70 µm cell strainer (Greiner Bio-One). For the SC, both enzymatic digestion, using collagenase D (500 µg/ml, Roche Diagnostics GmbH) and DNase I (40 U/ml, Roche Diagnostics GmbH), and mechanical dissociation were performed, followed by a Percoll gradient (GE Heatlhcare). Next, isolated cells were stained for 15 min in 1 × PBS containing 2% FCS and 0.1% azide using following antibodies (all purchased from BioLegend): CD19 BV650 (clone 6D5, 1:400), CD11b PerCP-Cy5.5 (clone M1/70, 1:400), CD11c FITC (clone N418, 1:200), Ly6G APC-Fire810 (clone 1A8, 1:200), NK1.1 BV605 (clone PK136, 1:250), CD3 PE-Cy5 (clone 17A2, 1:200), I-A/I-E PerCP-Fire806 (clone M5/114.15.2, 1:100), CD5 APC-Fire750 (clone 53–7.3, 1:400), CD45 Alexa Fluor 700 (clone 30-F11, 1:400), CD4 BV510 (clone RM4-5, 1:100), CD8a Spark blue 574 (clone 53–6.7, 1:200), CD62L PE-Dazzle 594 (clone MEL-14, 1:400), Ly6C BV785 (clone HK1.4, 1:500), F4/80 APC (clone BM8, 1:200), P2RY12 BV421 (clone S16007D, 1:200), CD43 PE-Cy7 (clone S11, 1:200). Dead cells were excluded by incubation with Zombie NIR (1:1000). Cells were acquired on an Aurora spectral flow cytometer (Cytek Biosciences). The gating strategy is depicted in Fig. S1.

### Statistical analysis

Data are shown as boxplots with individual values, where the middle line represents the median and the box displays first and third quartiles, with whiskers extending to minimum and maximum values. Statistical tests based on three independent experiments were performed in GraphPad Prism v10.02 (GraphPad Software, La Jolla, USA), except for RNA-seq data analysis, which was a single experiment and implemented in R (v4.3.2) using RStudio (v2023.12.1). Area under the curve (AUC) values for EAE clinical scores were calculated using the trapezoidal method. Longitudinal EAE clinical scores were analyzed over the treatment period using a two-way repeated-measures ANOVA with time and treatment as factors, with Greenhouse–Geisser correction for violation of sphericity. The interaction term between time and treatment is reported.

In the case of a sample size > 5, outliers were identified and removed using the ROUT method (Q = 1%), which was applied to one sample in Fig. 4n (FLAPa) and one sample in Fig. 6l (FLAPa). Data normality was assessed using the Shapiro–Wilk test. For group comparison, we used an unpaired Student’s *t *test with Welch’s correction or a Mann–Whitney test in the case of non-parametric data. For grouped gene expression analysis, a two-way ANOVA with Sidak’s multiple comparisons correction was applied and the effect of treatment across the gene group is reported. In multiple (> 2) group comparisons, a one-way analysis of variance (ANOVA) with Tukey’s/Dunnett’s (paired) multiple comparisons test was applied. In the case of non-normally distributed data, the Kruskal–Wallis or Friedman test (paired) was used, followed by Dunn’s post hoc analysis. Analysis details are stated in figure legends, accompanied by corresponding *p *values, of which *p* < 0.05 (red) was considered statistically significant.

## Results

### Increased LTB_4_ levels in MS WM lesions

**Fig. 1:**
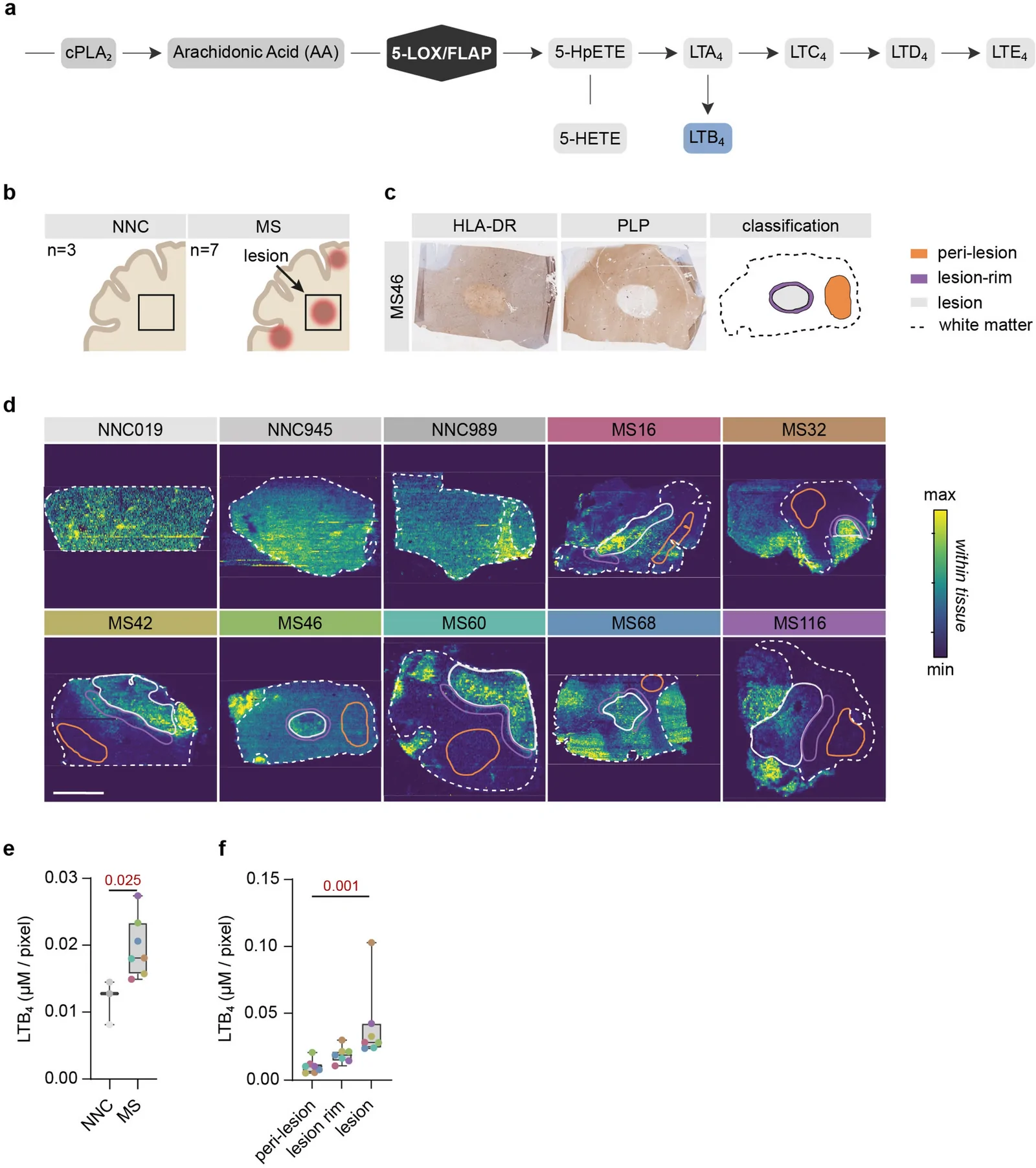
Increased LTB_4_ levels in MS lesion area.** a** Schematic overview of LTB_4_ (highlighted in blue) biosynthesis through the 5-LOX/FLAP pathway. **b** Three non-neurological controls (NNC) and seven MS post-mortem brain tissues were used **c** ROIs were assessed based on HLA-DR and PLP reactivity. For each MS sample, the white matter was outlined with a dotted line and a lesion ROI (grey), adjacent lesion rim (purple) and distant peri-lesion ROI (orange) were defined; scale bar 5 µm. **d** Ion images of LTB_4_ [M + ^107^Ag]^+^ were constructed by visualizing *m/z* 443.1346 normalized pixel-by-pixel to internal standard LTB_4_ in human non-neurological controls (NNC) and MS patients. The spatial distribution of LTB_4_ is visualized by min–max concentration scale within each single ion image; hence, the image scales are not related to each other. For each image, the lesion, rim, and peri-lesion ROI used for quantification are indicated; scale bar 5 µm. **e** LTB_4_ concentrations in white matter MS tissue compared to NNC. LTB_4_ signal intensities were extracted as average pixel intensity per tissue and normalized to the internal standard of LTB_4_-d_4_ to yield average detected concentration per pixel (µM/pixel). Data points are colored corresponding to tissues from different human donors depicted in panel (d). **f** Paired analysis of LTB_4_ concentration based on classification (one representative ROI) within MS tissues. Each colored dot presents one donor. Data are represented as median ± quartiles; whiskers extend to minimum and maximum. Data have been statistically tested for two groups by a paired *t *test with Welch’s correction. For three groups, a Friedman test (paired) for non-normally distributed data and Dunn’s post hoc analysis was applied. Exact *p *values are reported and statistical significance is set at *p* < 0.05 (red)

5-LOX and FLAP are mainly responsible for the two-step conversion of AA to LTA_4_, which is the precursor for LTB_4_ (Fig. 1a) [74]. Our previous work revealed lower AA levels in MS tissue compared to controls [21], suggesting its conversion into elevated downstream metabolite levels. To spatially map LTB_4_ levels in MS WM lesions, we histologically classified human post-mortem brain tissues from NNCs (*n* = 3) (Table 1) and MS WM lesions (*n* = 7) (Table 1**)** using PLP and HLA-DR immunoreactivity (Fig. 1b). This resulted in a total WM tissue area outline per donor, with three additional ROIs within the MS tissues, corresponding to the lesion, lesion rim, and peri-lesion (Fig. 1c). We then applied silver-doped PA nano-DESI MSI to visualize local LTB_4_ concentrations in human brain tissues. Ion images, visualizing relative LTB_4_ signal intensity within tissue sections, revealed profound regional heterogeneity of LTB_4_ concentrations in tissue sections with visible areas of higher intensity. While these high intensity regions partially localize to grey matter, which was excluded from our analysis, they are also visible within the WM whole tissue outlines of the MS cases, while NNC showed less variation (Fig. 1d). The mean detected LTB_4_ concentration [µM/pixel] per tissue section showed an increase in MS tissue compared to NNC (*p* = 0.025) (Fig. 1e). Paired analysis within MS tissue showed that LTB_4_ levels were increased in the lesion area compared to the peri-lesion area (*p* = 0.001), while no significant differences were observed in the lesion rim compared to the lesion areas (Fig. 1f). Together, these findings suggest that LTB_4_ shows a spatially heterogeneous distribution within MS WM tissue, with a relative enrichment within MS lesion areas.

### FLAP expression is restricted to microglia and induced in MS WM lesions

**Fig. 2:**
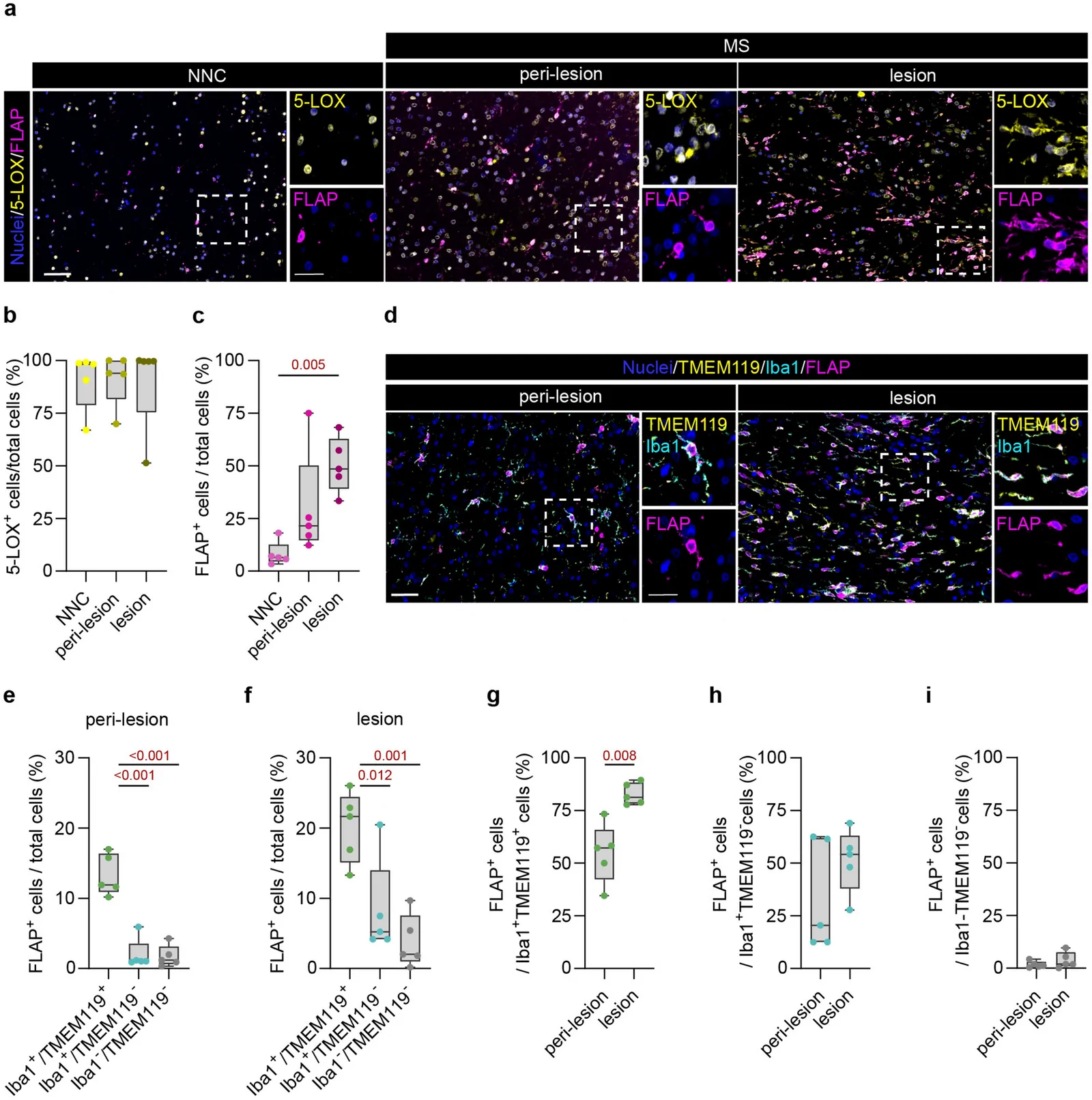
Increased microglial FLAP expression in MS white matter lesions. **a** Representative images of 5-LOX (yellow), and FLAP (magenta) immunoreactivity in white matter of non-neurological controls and mixed active/inactive MS WM lesions (peri-lesion and lesion). Panels show outlined excerpts at higher magnification; scale bar outset: 50 µm, inset 100 µm. **b–c** Quantification of 5-LOX^+^ cells (b) and FLAP^+^ cells (c) within the total cell population in NNC and MS tissues (*n* = 5). **d** Representative immunohistochemical images of TMEM119 (yellow), IBA1 (cyan), and FLAP (magenta) in MS tissue (peri-lesion and lesion). Panels show outlined excerpts at a higher magnification; scale bar outset 50 µm, inset 100 µm. **e–f** Quantification of FLAP^+^Iba1^+^TMEM119^+^ (green), FLAP^+^Iba1^+^TMEM119^−^(cyan), and FLAP^+^Iba1-TMEM119^−^ (grey) cells within the total cell population in peri-lesion (e) and lesion (f) area in MS brain tissue (*n* = 5). **g–i** Quantification of FLAP^+^ cells within the Iba1^+^TMEM119^+^(g), Iba1^+^TMEM119^−^ (h), and Iba1^−^TMEM119^−^ (i) cell population. Data are shown as box plots with median ± quartiles; whiskers extend to minimum and maximum. When three groups were compared, data were statistically tested by one-way ANOVA with Tukey’s correction. For two groups, an unpaired *t *test with Welch’s correction was used. Exact *p *values are reported and statistical significance is set at *p* < 0.05 (red)

The observed increase of LTB_4_ in MS lesion areas suggests its local biosynthesis via 5-LOX and FLAP. To verify this hypothesis and to identify the cellular producers of LTB_4_, we used human brain tissues from NNCs (*n* = 5) and MS (*n* = 5) for immunohistochemistry (IHC) to assess the cellular localization of the respective LTB_4_-biosynthetic enzymes. We observed that 5-LOX is highly abundant in WM brain tissue, with no differences in percentage of cells positive for 5-LOX between NNC, MS peri-lesion, and lesion areas (Fig. 2a, b). In contrast, FLAP was lowly expressed in NNC and profoundly increased in MS tissue, with a significantly higher percentage of cells positive for FLAP in MS WM lesions compared to NNC (*p* = 0.005) (Fig. 2b, c). In line with this, when using MS tissue lysates to quantify 5-LOX and FLAP gene expression using RT-qPCR, transcripts of *ALOX5* (encoding 5-LOX) did not show significant differences between NNC and MS, while *ALOX5AP* (encoding FLAP) transcripts were higher in the MS tissues compared to NNC (*p* = 0.045) (Fig. S2a, b).

To define the cell types harboring the observed FLAP immunoreactivity, we next performed additional immunohistochemical analysis on MS tissues (*n* = 5) by co-staining for FLAP with IBA1 (expressed by both microglia and infiltrating macrophages) and TMEM119 (microglia-specific marker) (Fig. 2d). Interestingly, we observed that the majority of FLAP^+^ cells were TMEM119^+^Iba1^+^ (microglia), while the TMEM119^−^Iba1^+^ (macrophages) and TMEM119^−^Iba1^−^ (remaining cells) populations demonstrated low FLAP expression in both peri-lesion (*p* < 0.001; *p* < 0.001, respectively) and lesion (*p* = 0.001; *p* = 0.012, respectively) areas (Fig. 2d–f). Additionally, when fractioning into the aforementioned populations of TMEM119^+^Iba1^+^, TMEM119^−^Iba1^+^, and TMEM119^−^Iba1^−^, the percentage of FLAP^+^ cells out of all TMEM119^+^Iba1^+^ cells, representing the microglial population, was higher in the lesion area compared to peri-lesion (*p* = 0.008) (Fig. 2g). On the other hand, no difference in percentage of FLAP^+^ cells comparing peri-lesion and lesion was found within TMEM119^−^Iba1^+^ and TMEM119^−^Iba1^−^ populations (Fig. 2h, i). Together, these findings suggest that MS WM lesions harbor an increased number of FLAP^+^ cells, which could be connected to increased FLAP abundance in the microglial population.

### FLAP antagonism in microglia reduces LTB_4_ biosynthesis and decreases inflammatory pathways in homeostatic cells

**Fig. 3:**
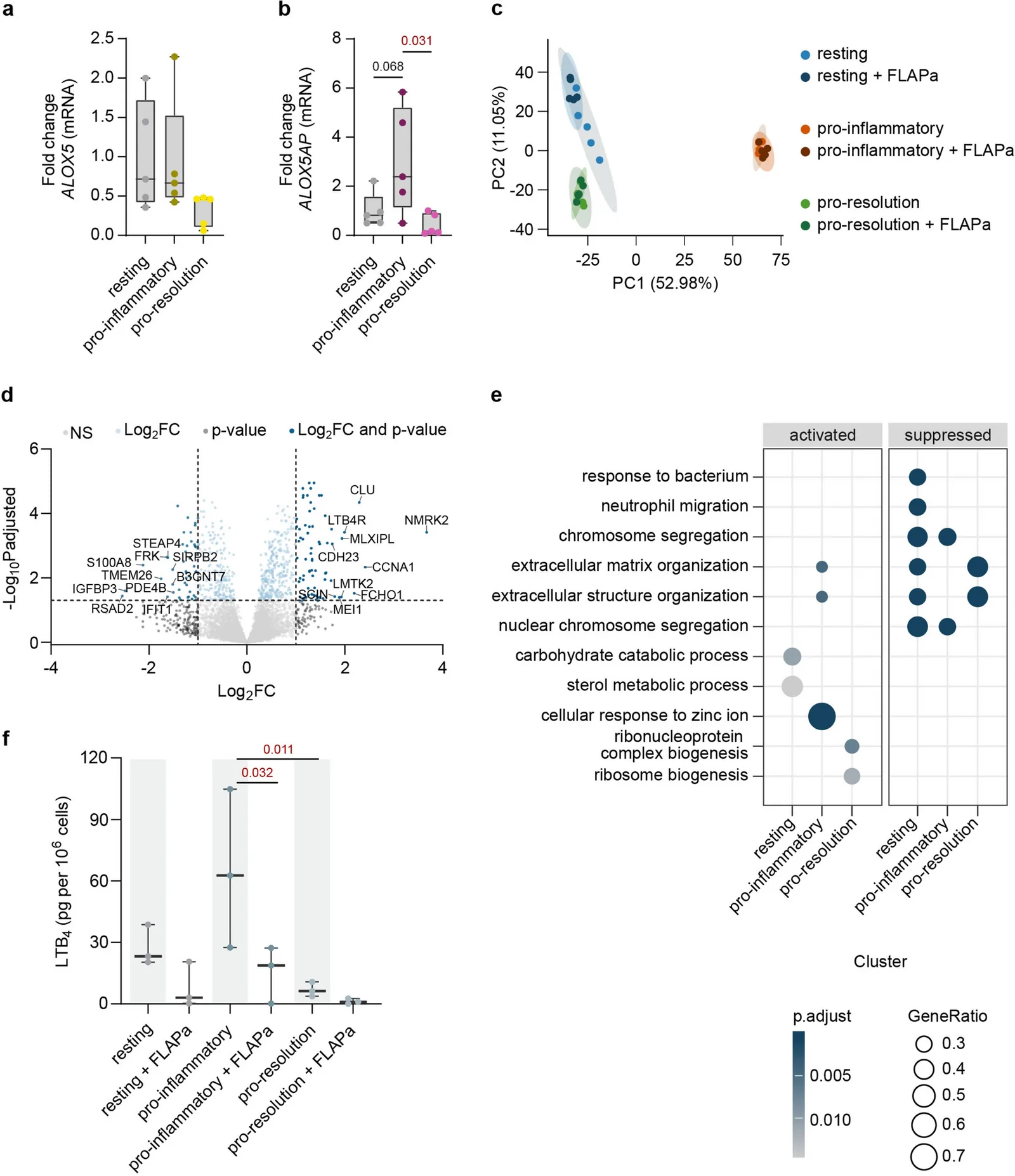
FLAP-mediated LTB_4_ production can be inhibited by FLAP antagonism in microglia while marginally impacting gene expression. **a–b** mRNA expression of *ALOX5* (a) and *ALOX5AP* (b) pro-inflammatory (LPS/IFNγ for 24 h), and pro-resolution (IL-4/IL-13 for 48 h) compared to resting hiPSC-derived microglia. **c** PCA plot of RNA-seq data of resting, pro-inflammatory, and pro-resolution microglia treated for 24 h with vehicle or FLAPa (fiboflapon, 0.5 µM). **d** Volcano plot representing significant DEGs comparing FLAPa treatment versus vehicle in resting microglia with respect to -log_10_ p adjusted in the *y*-axis and log_2_ fold change in the *x*-axis. **e** Enrichment GO terms from GSEA in resting, pro-inflammatory, and pro-resolution microglia treated with FLAPa (fiboflapon, 0.5 µM) compared to vehicle-treated cells. **f** LTB_4_ concentrations in resting, pro-inflammatory, and pro-resolution microglia. The LTB_4_ production was induced by 15 min incubation with Ca^2+^ and calcium ionophore in the presence of vehicle or FLAPa (fiboflapon, 0.5 µM) treatment. The experiment was done with one human donor and three (f)/five (a, b) independent differentiations, or five technical replicates (c). Data are shown as box plots with individual data points and median ± quartiles; whiskers extend to minimum and maximum. When three (a, b) or six (f) groups were compared, data were statistically tested by one-way ANOVA with Dunnett’s correction and Dunn’s post hoc analysis. Exact *p *values are reported and statistical significance is set at *p* < 0.05 (red)

Since FLAP was increased in microglia in human MS WM lesions, we further investigated the regulation and function of 5-LOX and FLAP in human microglia. For this, hiPSC-derived microglia were cultured under resting, pro-inflammatory (LPS/IFNγ for 24 h) and pro-resolving (IL-4/IL-13 for 48 h) conditions. Consistent with our observations in human brain tissue, *ALOX5* (encoding 5-LOX) transcripts did not differ between conditions, whereas *ALOX5AP* (encoding FLAP) transcript levels were highest under pro-inflammatory conditions, being significantly increased compared to pro-resolution conditions *(p* = 0.031), but not to resting conditions, despite a trend towards higher expression (*p* = 0.068) (Fig. 3a, b). In order to assess potential effects of FLAP interference on microglia phenotypes, we treated the conditioned hiPSC-derived microglia with the selective FLAP antagonist fiboflapon (FLAPa) or vehicle for 24 h and conducted bulk RNA sequencing [41, 67]. We detected 10,713 genes in total, and PCA plots reveal a clear separation of resting, pro-inflammatory and pro-resolution microglial phenotypes. However, as indicated in the PCA, we observed no major effects of FLAPa on overall microglia phenotype skewing, highlighted by the vehicle and FLAPa clusters overlapping within all three conditions (Fig. 3c). In line with this, when modeling the comparison of FLAPa to vehicle samples, we did not identify any differentially expressed genes (DEGs) under pro-inflammatory and pro-resolution conditions (Fig. S3**a, b**). On the contrary, the resting condition of hiPSC-derived microglia showed 121 DEGs (72 upregulated; 49 downregulated) upon FLAPa (Fig. 3d**; **Table S1). Pathway analysis based on gene set enrichment analysis (GSEA) revealed a suppression of infection- and inflammation-related gene ontology (GO) terms, such as response to bacterium and neutrophil migration, as a result of FLAPa treatment under resting conditions, suggesting an overall anti-inflammatory effect of FLAPa on homeostatic microglia, while not exerting any major transcriptional effects on the pro-inflammatory or pro-resolving phenotypes (Fig. 3e**; **Table S2).

In addition to phenotypic alterations, we also assessed the functional activity of the 5-LOX/FLAP pathway and the effect of FLAPa by quantifying LM biosynthesis using a targeted high-performance liquid chromatography–tandem mass spectrometry approach. As LTB_4_ is a short-lived molecule, we treated microglia after culturing them in resting, pro-inflammatory (LPS/IFNγ for 24 h) or pro-resolving (IL-4/IL-13 for 48 h) conditions with Ca^2+^ and calcium ionophore A23187 for 15 min to induce the activation of phospholipase A_2_, followed by the release of AA and facilitating subsequent LTB_4_ biosynthesis [32]. As expected, we observed the highest LTB_4_ levels in the pro-inflammatory microglia condition, which was significantly increased when compared to the pro-resolving condition (*p* = 0.011). Moreover, LTB_4_ levels in pro-inflammatory microglia were significantly reduced when FLAPa was applied (*p* = 0.032) without affecting the sum of COX-products, indicating specific antagonism of the 5-LOX/FLAP pathway by FLAPa in microglia. (Fig. 3f; Fig. S4a).

### FLAP antagonism ameliorates neuroinflammation by reducing local LTB_4_ levels

To assess whether the observed increase in FLAP in human MS tissue translates to the EAE in vivo model, we first used EAE SC lysates to quantify local *Alox5* and *Alox5ap* gene expression using RT-qPCR. In line with our findings in human brain tissue, we observed a significant increase in *Alox5ap* (encoding FLAP) expression in EAE mouse SC tissue during the chronic disease stage (day 28 post-immunization) of disease (*p* < 0.001) compared to control SC tissue, while no changes were observed in *Alox5* (encoding 5-LOX) (Fig. S5a, b).

**Fig. 4:**
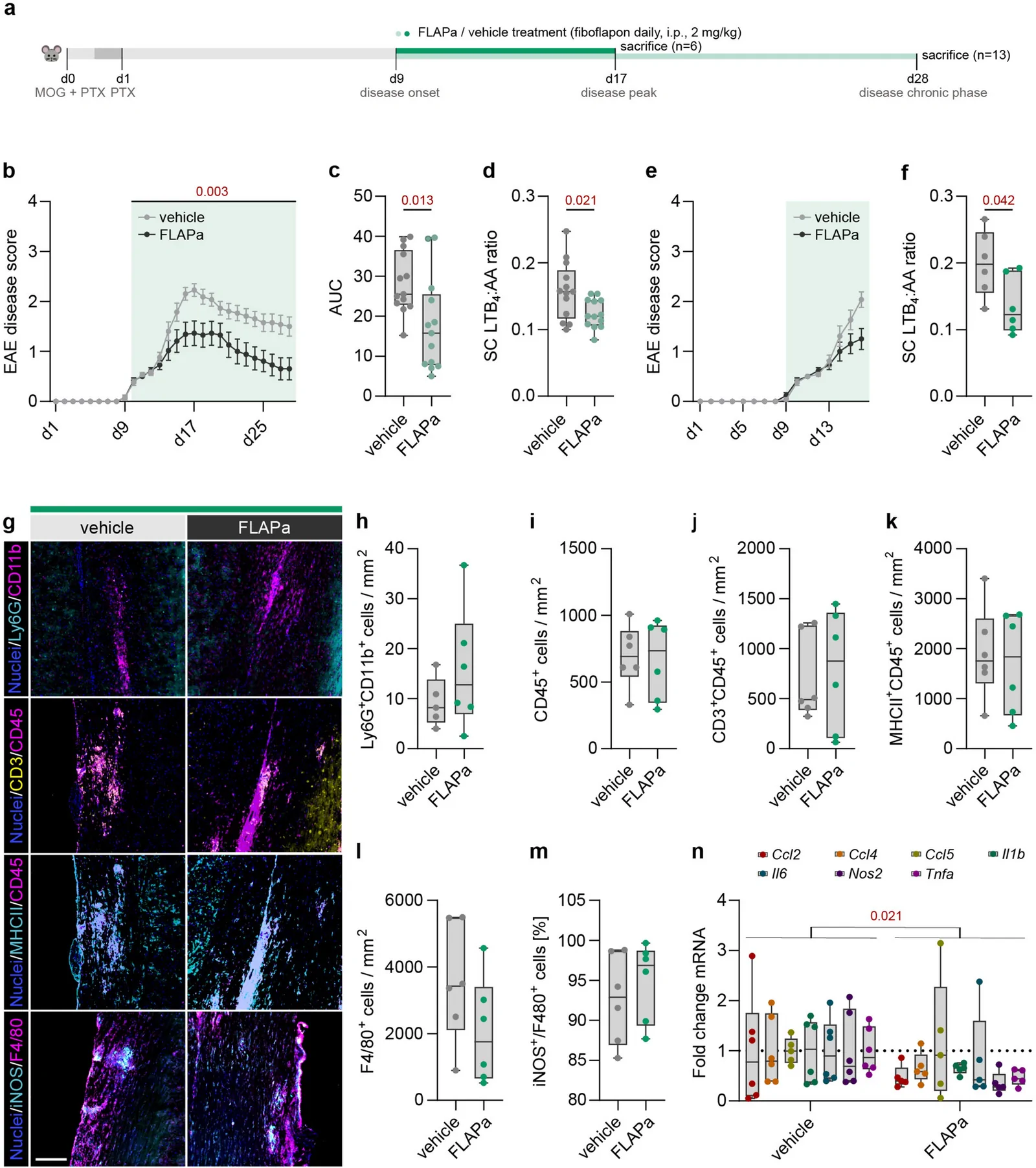
Pharmacological FLAP antagonism reduces neuroinflammation in the EAE model.** a** Schematic overview of EAE disease course and treatment paradigm. **b** Disease scores of EAE mice treated 9 days post-immunization with vehicle or FLAPa (fiboflapon 2 mg/kg, i.p., daily, *n* = 13 per group) (prophylactic). The shaded area indicates the treatment period, analyzed by two-way repeated-measures ANOVA with Greenhouse–Geisser correction. The interaction term between time and treatment is reported. **c** Area under the curve (AUC) quantification of EAE scores from panel (b). **d** LTB_4_ to arachidonic acid (AA) ratio in the spinal cords (SC) of treated EAE animals from panel B. **e** Disease scores of EAE mice treated 9 days post-immunization with vehicle or FLAPa (fiboflapon 2 mg/kg, i.p., daily, *n* = 13 per group) and sacrificed at the peak of disease (d17, *n* = 6 per group) for SC tissue characterization. **f** LTB_4_ to AA ratio in the SC of treated EAE animals from panel E. **g** Representative immunohistochemical staining for Ly6G/CD11b, CD3/CD45, MHCII/CD45, and F4/80/iNOS on SC tissue obtained from EAE animals treated with vehicle or FLAPa in the prophylactic setting and sacrificed at the peak of the disease; scale bar 200 µm. **h–k** Quantification of Ly6G^+^/CD11b^+^ cells (h, neutrophils), total CD45^+^ cells (i, immune cells), CD3^+^CD45^+^ cells (j, T cells), MHCII^+^CD45^+^ cells (k, activated macrophages/microglia) comparing vehicle-treated or FLAPa-treated EAE mice sacrificed at peak. **l-m** Quantification of total F4/80^+^ cells (l) and percentage of iNOS^+^ cells within the F4/80^+^ population (m) comparing vehicle-treated or FLAPa-treated EAE mice sacrificed at peak. **n** Quantitative PCR was used to determine mRNA levels of the pro-inflammatory genes *Ccl2*, *Ccl4*, *Ccl5*, *Il1b*, *Il6*, *Nos2*, and *Tnfa*. Data are shown as box plots with median ± quartiles; whiskers extend to minimum and maximum. For two groups, an unpaired *t *test with Welch’s correction was used (c, d, f, h-m) or the row factor was calculated using a two-way ANOVA with Sidak’s correction (n). Exact *p *values are reported and statistical significance is set at *p* < 0.05 (red)

To reveal whether FLAP antagonism influences the disease course of EAE, we applied the well-characterized and selective FLAP antagonist fiboflapon in the EAE mouse model. To confirm the CNS presence of fiboflapon during FLAPa treatment, LC–MS analysis was performed. Using brain tissue from EAE mice, fiboflapon could be detected based on accurate mass and retention time consistent with a reference standard, indicating that fiboflapon is present in the brain following systemic administration (Fig. S6a, b). For disease monitoring, EAE-induced mice were treated from disease onset (day 9 post-immunization, prophylactic) while being monitored and sacrificed at the peak of disease (day 17) or at the chronic phase of disease (day 28) (Fig. 4a). Overall, a decrease in body weight was observed after disease induction, which recovered in the chronic phase (Fig. S7a). Importantly, the group treated from disease onset until the chronic phase of disease displayed a significant amelioration in EAE severity over time, as shown by longitudinal analysis of clinical scores revealing a significant time × treatment interaction (d9–28: *p* = 0.003) (Fig. 4b). This was reflected by a reduced area under the curve in the FLAPa-treated group compared to vehicle (*p* = 0.013) (Fig. 4c). The group that was sacrificed at the peak of disease showed a similar disease progression and body weight pattern (Fig. 4e; Fig. S7b). To determine whether the reduced disease severity observed following FLAP antagonism (FLAPa) was associated with altered lipid mediator (LM biosynthesis) production, SCs were isolated for bulk LM analysis. The LTB_4_:AA concentration ratio was used as a measure of LTB_4_ production relative to the availability of its substrate, arachidonic acid (AA), thereby serving as a functional readout of 5-LOX/FLAP pathway activity and the impact of FLAPa treatment. Consistent with pathway inhibition, SC-derived LTB_4_:AA ratios were significantly reduced in FLAPa-treated animals compared with vehicle-treated controls, both at peak of disease and during the chronic phase of EAE (*p* = 0.042; *p* = 0.012, respectively) (Fig. 4d, f). Remaining 5-LOX products:AA concentration ratios were unaffected by FLAPa, aside from a reduction of 5-HETE:AA ratio under FLAPa treatment at the peak of disease (*p* = < 0.001) (Fig. S7c, e). Of note, absolute concentrations of detected 5-LOX products, including LTB_4_, showed no differences between groups, with the exception of 5-HETE, which was decreased upon FLAPa treatment (*p* < 0.001) at the peak of disease (Fig. S7d, f). Peripheral LTB_4_ levels measured in plasma were not detectable both at the peak of disease and the chronic stage (data not shown).

EAE clinical signs are driven by immune cell infiltration into the SC [80]. To assess whether FLAPa affects this pathological process, we next quantified immune cell presence using immunofluorescent analysis on longitudinal SC sections from EAE mice. Both vehicle- and FLAPa-treated groups showed EAE lesions at the peak and chronic phase of the disease, consisting of clusters of CD45^+^ immune cells in the white matter, composed of T cells (CD3^+^CD45^+^) as well as activated macrophages/microglia (MHCII^+^CD45^+^; F4/80^+^iNOS^+^) and a small population of neutrophils (CD11b^+^Ly6G^+^) (Fig. 4g; Fig. S8a). Quantification and comparison of FLAPa-treated mice to vehicle controls at the peak of disease showed no clear differences in the number of neutrophils, total number of immune cells, T cells or activated macrophages/microglia based on MHCII^+^CD45^+^ immunoreactivity (Fig. 4h–k). Additional immunostainings showed no significant differences in total number of microglia/macrophages (F4/80^+^) upon FLAPa treatment, as well no changes in the microglia/macrophage subpopulation that display an activated phenotype (F4/80^+^iNOS^+^) (Fig. 4l, m). At the chronic stage of disease, also no differences in SC immune cell populations were detected (Fig. S8b–g).

Next to immune cells, we also assessed the potential effect of FLAPa on local gene expression of pro-inflammatory protein mediators (*Ccl2, Ccl4, Ccl5, Il1b, Il6, Nos2, Tnfa*) in SC tissue lysates from vehicle- and FLAPa-treated mice. Overall, a reduction in inflammatory gene expression was observed after FLAPa treatment compared to vehicle at the peak of disease (*p* = 0.021), while at the chronic stage, FLAPa showed no differences (Fig. 4n, Fig S8h). Together, these findings show that FLAPa ameliorates EAE, by reducing LTB_4_ production defined as its concentration ratio to AA and decreasing pro-inflammatory markers but not absolute amounts of SC-infiltrated immune cells.

### FLAP antagonism reduces inflammatory monocytes within the SC while largely preserving overall immune cell composition

**Fig. 5:**
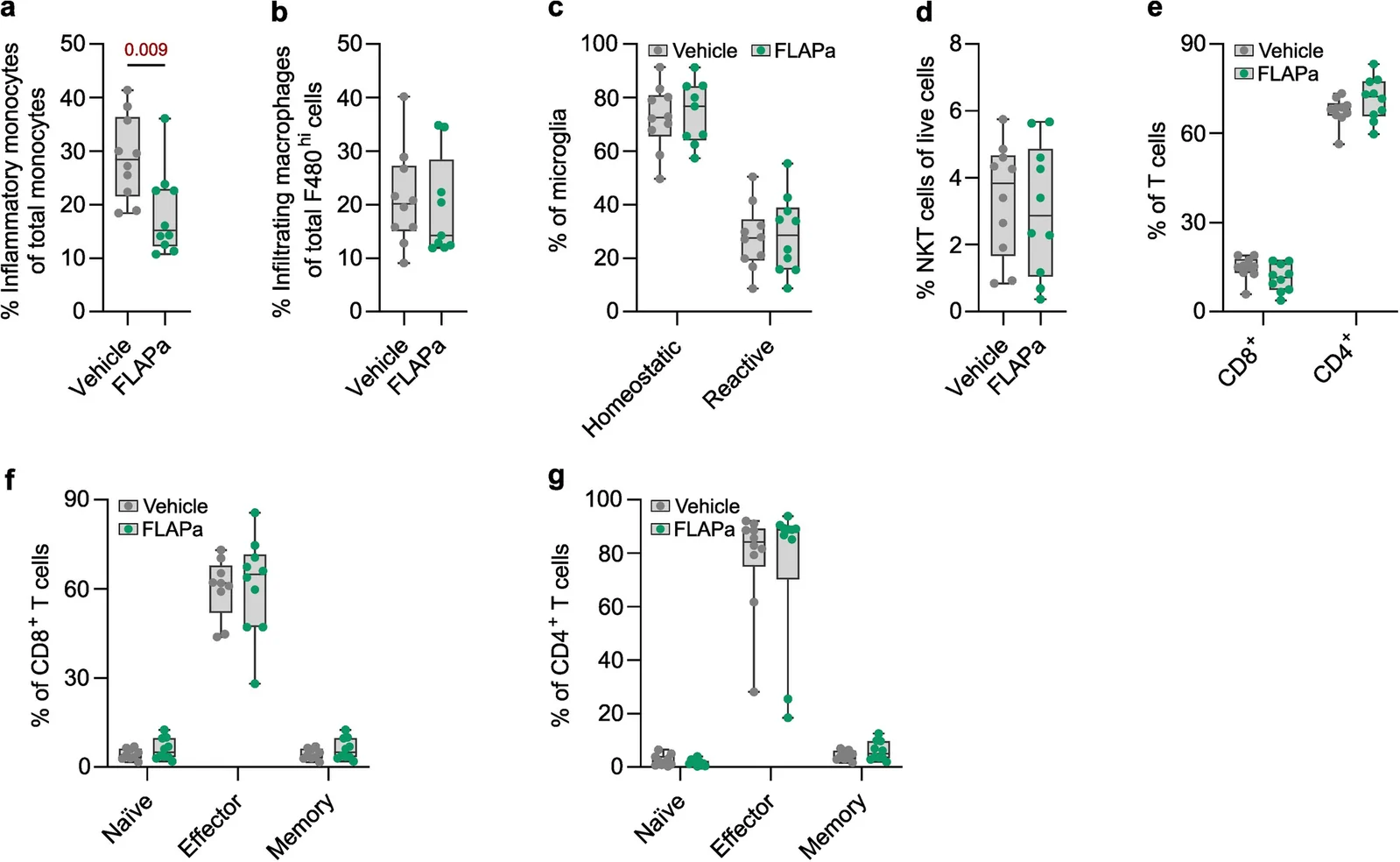
Pharmacological FLAP antagonism reduces inflammatory monocyte abundance in the spinal cord of EAE mice.** a** Inflammatory monocytes as percentage of total monocytes (*n* = 10 per group). **b** Infiltrating macrophages as percentage of F4/80^high^ cells (*n* = 10 per group). **c** Homeostatic (P2RY12^+^ CD45^low^) and reactive (P2RY12^+^ CD45^high^) microglial subpopulations of total microglia (P2RY12^+^) (*n* = 10 per group). **d** Percentage of natural killer T cells (NKT cells; NK1.1^+^ CD3^+^) within the viable cell population (*n* = 10 per group). **e–g** CD8^+^ and CD4^+^ T cell subsets (e) and their respective naïve (CD44^low^ CD62L^high^), effector (CD44^high^ CD62L^low^), and memory (CD44^high^ CD62L^high^) subpopulations (f-g) (*n* = 10 per group). Data are shown as box plots with median + quartiles; whiskers extend to minimum and maximum. For two groups, an unpaired *t *test with Welch’s correction was used (a, b, d) or the row factor was calculated using a two-way ANOVA with Sidak’s correction (c, e–g). Exact *p *values are reported and statistical significance is set at *p* < 0.05 (red)

To further assess the immune cell composition at the peak of disease (day 17 post-immunization), we performed flow cytometric analysis in EAE mice treated prophylactically (starting from day 9) with vehicle or FLAPa (Fig. S9a). In the SC, FLAPa treatment reduced the percentage of inflammatory monocytes among total monocytes (*p* = 0.009) (Fig. 5a), while the percentage of infiltrating macrophages within the F4/80^high^ population remained unchanged (Fig. 5b). Analysis of P2RY12^+^ microglia revealed no differences in the relative proportion of homeostatic (CD45^low^) and reactive (CD45^high^) subpopulations (Fig. 5c). NKT cells within the viable SC cell fraction were not significantly altered (Fig. 5d), and CD4^+^ and CD8^+^ T cell populations and their naïve (CD44^low^ CD62L^high^), effector (CD44^high^ CD62L^low^), and memory (CD44^high^ CD62L^high^) subsets were likewise unchanged (Fig. 5e–g).

To determine whether these findings reflected peripheral alterations, immune cell composition was next analyzed across blood and spleen. FLAPa treatment did not alter the overall distribution of leukocytes across compartments (Fig. S9**b**). Similarly, within the CD45^+^ leukocyte compartment, no broad changes in immune cell composition were observed in blood, spleen, or SC (Fig. S9**c–e**), although B cells were reduced in the spleen (*p* = 0.036) (Fig. S9d).

In-depth immunophenotyping showed no clear differences in the percentage of inflammatory monocytes among total monocytes in blood or spleen (Fig. S10a). NKT cell frequencies were reduced in the spleen (*p* = 0.046) as a result of FLAPa treatment, while in blood a trend towards reduction was observed (*p* = 0.104) (Fig. S10b). The overall proportions of CD8^+^ and CD4^+^ T cells were unchanged in blood and spleen (Fig. S10c, d). Within T-cell subsets, naïve CD8^+^ T cells were reduced in blood of FLAPa-treated mice (*p* = 0.008), whereas blood-derived naïve CD4^+^ T cell subsets showed a trend towards reduction (*p* = 0.082) (Fig. S10e, f). Effector and memory CD8^+^ and CD4^+^ T cell subsets in both blood and spleen were not significantly altered upon FLAPa (Fig. S10e, f. i, j). B cell and dendritic cell subpopulations were not significantly altered across compartments, although splenic B1 and B2 subsets showed a trend towards reduction (*p* = 0.077; *p* = 0.078, respectively) (Fig. S10g, h, k, l, m, n). Altogether, these data show that FLAP antagonism does not broadly reduce overall immune cell infiltration in the prophylactic setting in blood, spleen, and SC compartments, but instead induces selective changes in immune cell composition, most prominently reflected by a reduction in inflammatory monocytes in the SC during EAE.

### FLAP antagonism ameliorates neuroinflammation in a therapeutic setting

**Fig. 6:**
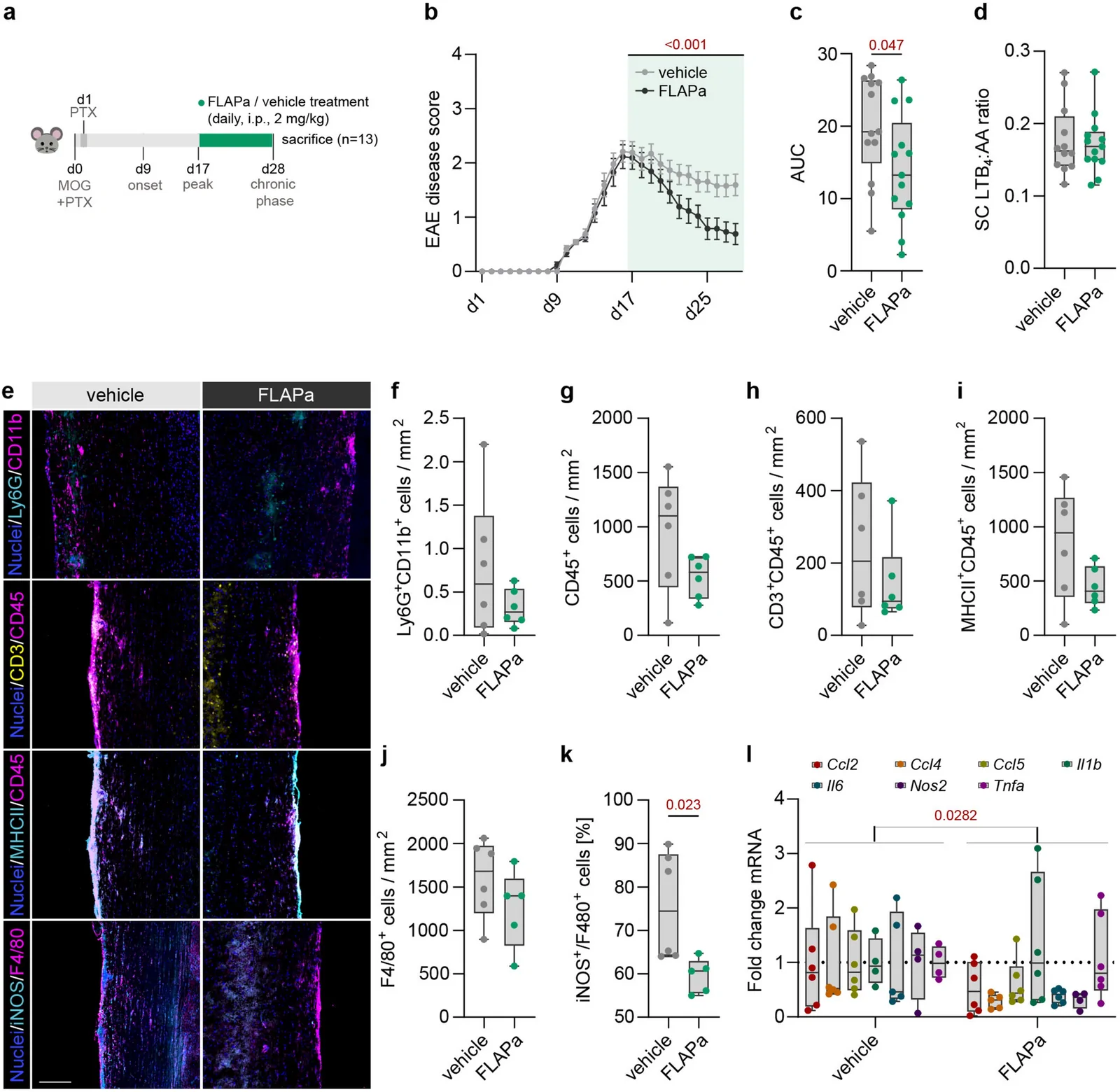
FLAP antagonism reduces EAE disease severity in a therapeutic setting. **a** Schematic overview of EAE disease course and treatment paradigm. **b** Disease scores of EAE mice treated from the peak of the disease, 17 days post-immunization with vehicle or FLAPa (fiboflapon 2 mg/kg, i.p., daily, *n* = 13 per group) (therapeutic). The shaded area indicates the treatment period, analyzed by two-way repeated-measures ANOVA with Greenhouse–Geisser correction. The interaction term between time and treatment is reported. **c** Area under the curve (AUC) quantification of EAE scores in panel B. **d** LTB_4_ to arachidonic acid (AA) ratio in the spinal cord (SC) of treated EAE animals from panel (b). **e** Representative immunohistochemical staining for Ly6G/CD11b, CD3/CD45, MHCII/CD45, and F4/80/iNOS on SC tissue obtained from EAE animals treated with vehicle or FLAPa in the therapeutic setting; scale bar 200 µm. **f–i** Quantification of Ly6G^+^/CD11b^+^ cells (f, neutrophils), total CD45^+^ cells (g), CD3^+^CD45^+^ cells (h, T cells), MHCII^+^CD45^+^ cells (i, activated macrophages/microglia) between vehicle-treated or FLAPa-treated EAE mice. **j–k** Quantification of total F4/80^+^ cells (j) and percentage of iNOS^+^ cells within the F4/80 population (k). **e** Quantitative PCR was used to determine mRNA levels of the pro-inflammatory genes *Ccl2*, *Ccl4*, *Ccl5*, *Il1b*, *Il6*, *Nos2*, and *Tnfa* to compare inflammation between treatment groups. Data are shown as box plots with median + quartiles; whiskers extend to minimum and maximum. For two groups, an unpaired *t *test with Welch’s correction was used (c-k) or the row factor was calculated using a two-way ANOVA with Sidak’s correction (l). Exact *p *values are reported and statistical significance is set at *p* < 0.05 (red)

To assess whether FLAP antagonism in EAE is also beneficial in a therapeutic setting, we next treated EAE mice with FLAPa or vehicle from the peak of disease (day 17 post-immunization) until sacrifice at the chronic phase (day 28) (Fig. 6a). Mice overall showed a decrease in body weight after disease induction (Fig. S11a). Therapeutical FLAPa markedly reduced the clinical signs of EAE over time, as demonstrated by a significant time × treatment interaction (d17–28: *p* < 0.001) (Fig. 6b). Consistently, this was reflected by a reduced area under the curve compared to vehicle-treated mice (*p* = 0.047) (Fig. 6c). The SC samples, isolated at the chronic phase of the disease, surprisingly showed no differences in LTB_4_:AA and remaining detected 5-LOX product:AA concentration ratios when comparing the FLAPa-treated group to the vehicle-treated group, except for a decrease in 5-HETE:AA (*p* < 0.001) (Fig. 6d; Fig. S11b). Of note, absolute 5-LOX product concentrations displayed no significant differences between groups (Fig. S11c). Additionally, peripheral LTB_4_ levels were not detectable in plasma (data not shown). The number of neutrophils, total immune cells, T cells and activated macrophages/microglia based on MHCII^+^CD45^+^ immunoreactivity did not differ between the vehicle- and FLAPa-treated group (Fig. 6e–i). Also, no difference in total microglia/macrophage number (F4/80^+^) was observed, while the activated subpopulation (F4/80^+^iNOS^+^) was decreased upon FLAPa treatment (*p* = 0.023) (Fig. 6j, k). The therapeutic effect and decrease in activated microglia/macrophages were also accompanied by an overall reduction of SC gene expression of pro-inflammatory protein mediators (*Ccl2, Ccl4, Ccl5, Il1b, Il6, Nos2, Tnfa*) after FLAPa treatment (*p* = 0.028) (Fig. 6l). Taken together, these results indicate that FLAPa also ameliorates EAE in a therapeutic setting, linked to a reduction in local macrophage/microglia activation and inflammation-related genes.

## Discussion

The chronic, unresolved nature of neuroinflammation in the CNS of PwMS presents a major therapeutic challenge, primarily due to an incomplete understanding of underlying dysregulated inflammatory and pro-resolving pathways. Here, we characterize the spatial distribution of LTB_4_ within WM MS lesions and identify lesion-associated increases in LTB_4_ accompanied by elevated microglial FLAP expression, consistent with the involvement of the FLAP-LTB_4_ axis. Additionally, we show that pharmacological antagonism of FLAP with fiboflapon suppresses LTB_4_ production in pro-inflammatory human iPSC-derived microglia and after prophylactic treatment in EAE SC. Furthermore, FLAP antagonism mitigates EAE disease severity in both prophylactic and therapeutic paradigms, underscoring the therapeutic potential of the FLAP/LTB_4_ axis for inflammatory CNS disorders.

Previously, other studies already demonstrated elevated LTB_4_ levels in the CSF of PwMS compared to healthy controls [34, 45, 46]. Additionally, AA-derived mediator production and *ALOX5AP* expression were both increased in damage-associated microglia in WM lesions [71]. Here, we define the spatial distribution of this LM within the MS lesion microenvironment. Using MSI on human WM from NNC and PwMS, we generated spatial maps of LTB_4_ abundance in and around MS lesions, revealing significantly increased LTB_4_ concentrations within lesion areas. Notably, in the same tissue cohort, we previously demonstrated a significant reduction in AA levels [21], suggesting enhanced downstream conversion of AA into bioactive LMs such as leukotrienes and prostaglandins. It is worth noting, however, that the tissue cohort is limited in size and consists of a variety of lesion types. Thus, any conclusions regarding lesion stage or activation would require further research in a more comprehensive cohort. Recently, similar lipid dynamics were observed in a lysolecithin-induced focal demyelination mouse model, where lesion homogenates showed a transient increase in free AA levels 14 days post-injection, followed by a decline, likely reflecting an inflammation-driven release and subsequent metabolic conversion into downstream LMs [24, 49]. Together, these findings indicate that reduced storage and enhanced metabolism of AA synergistically contribute to the lesion-specific depletion of AA in MS.

In this study, analysis of human MS tissue revealed upregulation of the LTB_4_ producing enzyme *ALOX5AP* (FLAP) by microglia, both at the transcriptional and at the protein level, predominantly within lesion areas, whereas *ALOX5*/5-LOX levels remained unaltered. This discrepancy suggests that 5-LOX expression may be less tightly coupled to inflammatory activation in the CNS than FLAP, which represents the more dynamically regulated and functionally relevant component of the LT biosynthetic machinery. In support of this, *ALOX5* and *ALOX5AP* are known to be regulated through overlapping, but not identical pathways. *ALOX5* expression relies on a Sp1/Egr-1 rich promoter that is further amplified by inflammatory transcription factors such as NF-κΒ, while *ALOX5AP* lacks the Sp1/Egr-1 hub and is governed by distinct promotor elements and a broader set of inflammation-responsive transcription factors such as NF-κΒ, c/EBP, and AP-1 [27, 52, 58, 59, 62, 63]. Additionally, *ALOX5AP* expression is known to be induced by pro-inflammatory stimuli such as TNF-α and LPS [52, 62] and its upregulation has been reported in activated microglia and macrophages in both MS lesions and animal models of neuroinflammation [29, 71]. Consistently, in the EAE SC and pro-inflammatory iPSC microglia, only *ALOX5AP* and not *ALOX5* mRNA levels were increased, mirroring its association with active inflammatory states. Together, these findings argue for FLAP as a key regulator of pro-inflammatory LM synthesis and highlight its potential as a therapeutic target to mitigate neuroinflammation.

Targeting FLAP provides a selective way to modulate 5-LOX activity, as FLAP is required for 5-LOX translocation and LT biosynthesis. The role of 5-LOX in MS is complex: EAE progression is exacerbated in 5-LOX–deficient mice, suggesting a protective function, yet pharmacological inhibition in cuprizone-treated mice attenuates neuroinflammation, motor dysfunction, and axonal damage without affecting demyelination [15, 85]. In human monocyte-derived macrophages, FLAP antagonism reduces 5-LOX product formation, like LTB_4_, without affecting the formation of specialized pro-resolving mediators that promote resolution [9, 32, 76]. Broad inhibition of 5-LOX, by contrast, suppresses both leukotriene and beneficial pro-resolving pathways and has been linked to adverse outcomes such as impaired resolution of inflammation and increased susceptibility to infection of pre-clinical models, highlighting the safety advantage of FLAP-focused strategies [3, 43]. Collectively, these findings indicate that FLAP modulation offers a more precise intervention than general 5-LOX inhibition, limiting LT-mediated inflammation while preserving basal 5-LOX function, which may enhance both efficacy and safety in neuroinflammatory contexts.

Pharmacological antagonism of FLAP has already been applied in numerous inflammatory diseases that show increased *ALOX5AP* expression in myeloid cells, including asthma and atherosclerosis, to reduce inflammation, with some inhibitors advancing successfully to clinical trials [11, 18, 51]. Here, we made use of the well-characterized and selective FLAP antagonist fiboflapon that prevents AA translocation and LT formation [41, 67]. Consistent with earlier work, our data show that the marked increase in LTB_4_ formation in pro-inflammatory iPSC microglia could effectively be reduced to baseline by FLAPa [9, 41, 67]. To investigate the effects of FLAP antagonism on gene expression, we performed RNA sequencing on vehicle- and FLAPa-treated iPSC microglia under resting, pro-inflammatory, and pro-resolution conditions. Surprisingly, only marginal transcriptional changes were detected following FLAPa, with the biggest differences in gene expression observed in resting microglia. This minor impact on gene expression likely reflects the mode of action of fiboflapon, which acts at the enzymatic level to control LTB_4_ production, rather than directly reprogramming microglial transcription. Moreover, the strong inflammatory stimuli applied in our model are not comparable to in vivo situations and may have induced a near-maximal pro-inflammatory response that is relatively insensitive to modulation of a single LM producing enzyme. Finally, compensatory pathways or post-transcriptional regulation could preserve the transcriptional signature despite changes in LTB_4_ production. Taken together, these findings suggest that FLAP antagonism primarily modulates the LM landscape and downstream paracrine signaling rather than broadly transcriptionally reprogramming activated microglia.

In the context of (neuro)inflammation, LTB_4_ is proposed primarily to originate from activated microglia/macrophages and infiltrating neutrophils [23, 25, 44]. Our ion images show that LTB_4_ concentrations peak in MS lesions. Consistently, FLAP expression is increased in microglia at the lesion site, suggesting that these might be a potent source of LTB_4_ in active MS WM lesions. However, we cannot rule out the possibility of additional LTB_4_ biosynthesis by other cell types or its leakage across the compromised blood–brain barrier.

LTB_4_ predominantly signals via the high-affinity receptor BLT1 and low-affinity receptor BLT2, promoting chemotaxis of lymphocytes, T-cell activation, and reactive oxygen species production [25, 37, 70, 84]. In addition, BLT1-deficient mice show a delay in EAE onset and reduced lymphocyte, neutrophil, and macrophage infiltration [39]. Given these receptor-mediated effects, it would be of considerable interest to determine whether FLAP antagonism also influences BLT1/2-dependent signaling in our experimental models. Although beyond the scope of this study, it would provide valuable information of the downstream mechanisms involved. Furthermore, the possibility that LTB_4_ may directly affect other CNS-resident cells such as endothelial cells, astrocytes, oligodendrocytes and neurons, and thereby influence disease progression, cannot be excluded and warrants investigation in future studies. In summary, LTB_4_ production at lesion cores may act as an amplifier of inflammation, raising the question whether FLAP antagonism can modulate neuroinflammation in vivo.

To address this question, we made use of two therapeutic paradigms in the EAE mouse model: treatment initiated at disease onset, and treatment initiated at the peak of the disease. In both settings, FLAPa improved clinical scores with a comparable magnitude, indicating that FLAPa remains effective even after disease onset. Interestingly, SC LTB_4_:AA ratios were only significantly reduced when treatment was initiated at disease onset, with the greatest reduction observed at the peak of the disease, indicating a reduced metabolization of AA into LTB_4_. In contrast, plasma LTB_4_ levels were not detectable in any group, despite prior studies showing reductions in systemic LTB_4_ following FLAPa with fiboflapon [2, 41]. Importantly, in these studies, the LTB_4_ biosynthesis was first stimulated using a calcium ionophore or zymosan. The absence of reduced plasma LTB_4_ levels may, therefore, be due to the timing of drug administration relative to transient LT bursts or potential compensatory systemic metabolic mechanisms [42, 44, 47]. While LTB_4_ can be increased locally in the inflamed EAE SC, systemic levels may be altered only transiently. Alternatively, plasma LTB_4_ levels may be buffered by compensatory metabolic pathways or rapid clearance, thereby masking peripheral changes despite robust suppression in the CNS [42]. In this case, early FLAPa coincides with a peak in CNS leukotriene biosynthesis, allowing maximal suppression of LTB_4_ accumulation, whereas later treatment may confer clinical benefit through non-LTB_4_ mechanisms such as reduced secondary lipid-mediator cascades or limiting local chemoattraction, even when bulk SC LTB_4_ levels are no longer different [25, 37, 39, 70].

Despite the robust clinical benefit, neither treatment strategy altered T cell, neutrophil or macrophage CNS infiltration. Notably, both prophylactic and therapeutic treatment induced a less inflammatory gene expression profile in the EAE SC. In the therapeutic setting, this was accompanied by a shift towards a less inflammatory microglia/macrophages phenotype at the protein level. Importantly, flow cytometric analyses in the prophylactic setting revealed more subtle but functionally relevant immunomodulatory effects. Specifically, FLAPa treatment reduced the abundance of inflammatory macrophages in the SC. Infiltrated monocyte-derived macrophages are well-established drivers of EAE progression [1, 86], with Ly6C^high^ monocytes relying on LTB_4_ signaling for CNS recruitment [19, 23, 36]. Beyond chemotaxis, LTB_4_ amplifies inflammatory transcriptional programs in monocytes via NF-kβ, AP-1, and MyD88-dependent signaling [57, 60, 66, 75], driving CCL2, Il1-β, Il-6, and TNF-α production [26, 54, 55, 65]. Diminished LTB_4_ may therefore limit monocyte CNS trafficking and attenuate downstream signaling, providing a basis for reduced inflammatory monocyte burden and suppressed SC cytokine/chemokine expression levels. Reduced splenic B cells are consistent with LTB_4_’s direct pro-proliferative effects on B lymphocytes [12, 81] while the decrease in NKT cells most likely reflects broader systemic immunomodulation rather than a direct LTB_4_-NKT axis, given the limited evidence for BLT1 expression on this population [56, 72].

These findings suggest that FLAPa may ameliorate disease severity through two complementary, timing-dependent routes: early treatment may mainly act by blunting the initial CNS-restricted wave of FLAP-dependent leukotriene production that amplifies myeloid activation, while later intervention might mainly act by reprogramming already activated microglia/macrophages towards a less inflammatory state without significantly altering either CNS or systemic LTB_4_ pools. This framework partially aligns with the well-established role of LTB_4_ as a chemotactic amplifier of immune-cell trafficking, the transient and spatially restricted nature of LT production within inflamed CNS tissues, and emerging evidence that FLAP governs additional 5-LOX-dependent lipid pathways beyond LTB_4_ alone [16, 25, 32, 37, 38, 70, 84]. In demyelinating and neuroinflammatory disorders, immune cells frequently accumulate at perivascular, meningeal, or subpial interfaces, and their migration into the parenchyma may require precise chemotactic cues, including LTB_4_ [28, 64]. By limiting FLAP-dependent LTB_4_ synthesis, FLAPa may attenuate recruitment and motility of peripheral immune cells, thereby ameliorating clinical disease scores even in the absence of extensive transcriptional or histological changes. Future studies are warranted to define these underlying changes.

Together, these data highlight that FLAPa provides substantial clinical benefit mechanisms that may not be fully captured by endpoint measurements of a single LM in either tissue or plasma, underscoring the importance of temporal and cell-specific lipid signaling in neuroinflammation.

Although FLAPa reduced LTB_4_ levels and ameliorated disease symptoms, changes in immune-cell infiltration and transcriptional profiles were modest. Additionally, the therapeutic efficacy was greatest when treatment was initiated early, suggesting that FLAP blockade alone may be insufficient to resolve established inflammation. While fiboflapon could be detected in the CNS following systemic administration, and reduced LTB_4_ levels were observed in the SC, a definitive assessment of CNS exposure and tissue distribution is warranted. Here, limited dosing, suboptimal CNS penetration or enhanced brain accumulation due to perturbed BBB integrity may have further constrained therapeutic benefit. Additionally, a potential contribution of peripheral effects cannot be excluded. Finally, while iPSC microglia provide a human-relevant platform, they may not fully recapitulate the complex cellular interactions and heterogeneity present in MS lesions. These limitations underscore the need for further studies to optimize FLAP-targeted strategies and to evaluate combinatorial approaches with existing therapies.

## Conclusion

To conclude, in the current research, we provide a spatial distribution of LTB_4_ in MS WM lesions, which was coupled to enhanced local microglial FLAP-LTB_4_ signaling. In vivo, FLAPa reduces SC LTB_4_ biosynthesis from AA, dampens inflammatory gene expression, and improves clinical EAE scores both in prophylactic and therapeutic settings, while selectively altering immune cell compositions, including a reduction in inflammatory monocytes in the SC. Collectively, these findings support a model in which local FLAP-LTB_4_ signaling amplifies neuroinflammation and highlights the therapeutic potential of inhibiting this axis as a promising strategy to combat neuroinflammation.

## Supplementary Information

Below is the link to the electronic supplementary material.

Supplementary file1 (JPG 2260 KB)

Supplementary file2 (TIF 1352 KB)

Supplementary file3 (TIF 3521 KB)

Supplementary file4 (TIF 1966 KB)

Supplementary file5 (TIF 1569 KB)

Supplementary file6 (JPG 328 KB)

Supplementary file7 (JPG 298 KB)

Supplementary file8 (TIF 9462 KB)

Supplementary file9 (JPG 631 KB)

Supplementary file10 (JPG 933 KB)

Supplementary file11 (TIF 7312 KB)

Supplementary file12 (XLSX 23 KB)

Supplementary file13 (XLSX 91 KB)

Supplementary file14 (DOCX 7513 KB)

## Data Availability

All data presented in this study are available from the corresponding author upon reasonable request.

## Abbreviations

AA: Arachidonic acid
AUC: Area under the curve
BLT: Leukotriene B receptor
CNS: Central nervous system
COX: Cyclooxygenase
CSF: Cerebrospinal fluid
DEG: Differentially expressed genes
EAE: Experimental autoimmune encephalomyelitis
FF: Formalin fixed
FLAP: 5-Lipoxygenase activating protein
FLAPa: FLAP antagonism
GO: Gene ontology
GSEA: Gene set enrichment analysis
HPLC: High-performance liquid chromatography
IFN: Interferon
IL: Interleukin
iPSC: Induced pluripotent stem cell
LC–MS: Liquid chromatography–mass spectrometry
LM: Lipid mediators
LOX: Lipoxygenase
LPS: Lipopolysaccharide
LTA_4_: Leukotriene A_4_
LTB_4_: Leukotriene B_4_
MS: Multiple sclerosis
MSI: Mass spectrometry imaging
NAWM: Normal-appearing white matter
NNC: Non-neurological control
NSS: Normal species serum
PA nano-DESI: Pneumatically assisted nanospray desorption electrospray ionization
PBS: Phosphate buffered saline
PCA: Principal component analysis
PLP: Proteolipid protein
PUFAs: Polyunsaturated fatty acids
PwMS: People with MS
ROI: Region of interest
RT: Room temperature
SC: Spinal cord
SPE: Solid-phase extraction
TMEM119: Transmembrane protein 119
WM: White matter
