# Correction: Microglial 5-LOX-activating protein antagonism alleviates leukotriene-driven neuroinflammation

**DOI:** 10.1007/s00401-026-03081-8

**Published:** 2026-09-04

**Authors:** Julia Konings, Fleur Mingneau, Serhii Chornyi, Cathrin E. Hansen, Rianne T. M. van der Burgt, Tessa G. van Elk, Laura Bolkaerts, Bo Batens, Gábor Tóth, Ingela Lanekoff, Frédéric M. Vaz, Alessio Cardilli, Marie Sels, Susanne M. A. van der Pol, Helga E. de Vries, Martin Giera, Oliver Werz, Sofie Kessels, Sanne G. S. Verberk, Jeroen F. J. Bogie, Merel Rijnsburger, Jerome J. A. Hendriks, Gijs Kooij

**Affiliations:** 1 Department of Molecular Cell Biology and Immunology, Amsterdam UMC Location Vrije Universiteit Amsterdam, De Boelelaan 1117, Amsterdam, The Netherlands; 2 Amsterdam Neuroscience, Amsterdam UMC, Amsterdam, The Netherlands; 3 MS Center Amsterdam, Amsterdam UMC Location VU Medical Center, Amsterdam, The Netherlands; 4 Department of Immunology and Infection, Biomedical Research Institute, Hasselt University, Diepenbeek, Belgium; 5 University MS Center, Pelt, Belgium; 6 Center for Proteomics and Metabolomics, Leiden University Medical Centre (LUMC), Leiden, The Netherlands; 7 Amsterdam Institute for Immunology & Infectious Diseases, Amsterdam, The Netherlands; 8 Department of Chemistry for Life Sciences, Uppsala University, Uppsala, Sweden; 9 Center for Excellence for the Chemical Mechanisms of Life, Uppsala University, Uppsala, Sweden; 10 Laboratory Genetic Metabolic Diseases, Amsterdam UMC, Amsterdam, The Netherlands; 11 Core Facility Metabolomics, Amsterdam UMC, Amsterdam, The Netherlands; 12 Department of Biochemistry, Cardiovascular Research Institute Maastricht, University of Maastricht, Maastricht, The Netherlands; 13 Department of Pharmaceutical/Medicinal Chemistry, Institute of Pharmacy, Friedrich Schiller University, Jena, Germany; 14 Jena Center for Soft Matter (JCSM), Friedrich Schiller University Jena, Jena, Germany; 15 Amsterdam Institute for Immunology and Infectious Diseases, Amsterdam UMC, Amsterdam, The Netherlands

**Correction: Acta Neuropathologica (2026) 152:16** **https://doi.org/10.1007/s00401-026-03055-w**

In the original version of this article, the given and family name of the author was incorrectly written as “Rianne T. M. van der Brugt” but the correct name should be “Rianne T. M. van der Burgt”.

The original article has been corrected.

