# Supplementary material for: Microglial 5-LOX-activating protein antagonism alleviates leukotriene-driven neuroinflammation
Source: Acta Neuropathol. 2026 Aug 4;152(1):16. doi: 10.1007/s00401-026-03055-w (PMC13437591; doi:10.1007/s00401-026-03055-w)
Supplement: Supplementary file 1 [file 401_2026_3055_MOESM14_ESM.docx]

**Supplementary figures and legends**

**Microglial 5-LOX-activating protein antagonism alleviates leukotriene-driven neuroinflammation**

Julia Konings^1,2,3*^, Fleur Mingneau^4,5*^, Serhii Chornyi^1,6^, Cathrin E. Hansen^1,2,3^, Rianne van der Brugt^1,2,7^, Tessa G. van Elk^1,2,3^, Laura Bolkaerts^4,5^, Bo Batens^4,5^, Gabor Toth^8,9^, Ingela Lanekoff^8,9^, Frédéric M. Vaz^10,11^, Alessio Cardilli^4,5^, Marie Sels^4,12^, Susanne M.A. van der Pol^1^, Helga E. de Vries^1,2,3^, Martin Giera^6^, Oliver Werz^13,14^, Sofie Kessels^4,5^, Sanne G.S. Verberk^4,5^, Jeroen F.J. Bogie^4,5^, Merel Rijnsburger^1,2,3^,Jerome J.A. Hendriks^4,5*^, Gijs Kooij^1,2,3,15*#^.

^*^ These authors contributed equally to this work

^1^	Amsterdam UMC location Vrije Universiteit Amsterdam, Department of Molecular Cell Biology and Immunology, De Boelelaan 1117, Amsterdam, The Netherlands

^2^	Amsterdam Neuroscience, Amsterdam UMC, Amsterdam, The Netherlands

^3^	MS Center Amsterdam, Amsterdam UMC Location VU Medical Center, Amsterdam, The Netherlands

^4^	Department of Immunology and Infection, Biomedical Research Institute, Hasselt University, Diepenbeek, Belgium

^5^	University MS Center, Pelt, Belgium

^6^	Leiden University Medical Centre (LUMC), Center for Proteomics and Metabolomics, Leiden, The Netherlands

^7^	Amsterdam institute for immunology & Infectious diseases, Amsterdam, The Netherlands

^8^	Department of Chemistry for Life Sciences, Uppsala University, Uppsala, Sweden

^9^	Center for Excellence for the Chemical Mechanisms of Life, Uppsala University, Uppsala, Sweden

^10^	Laboratory Genetic Metabolic Diseases, Amsterdam UMC, Amsterdam, the Netherlands

^11^	Core Facility Metabolomics, Amsterdam UMC, Amsterdam, the Netherlands

^12^	Department of Biochemistry, Cardiovascular Research Institute Maastricht, University of Maastricht, Maastricht, The Netherlands.

^13^	Department of Pharmaceutical/Medicinal Chemistry, Institute of Pharmacy, Friedrich Schiller University, Jena, Germany

^14^	Jena Center for Soft Matter (JCSM), Friedrich Schiller University Jena, Jena, Germany

^15^	Amsterdam Institute for Immunology and Infectious Diseases, Amsterdam UMC, Amsterdam, The Netherlands

^#^ Corresponding author: Gijs Kooij (email: g.kooij@amsterdamumc.nl, tel: +31 (0) 204448080)

**Figure S1 | Flow cytometry gating strategy for blood, splenocytes and spinal cord.** Gating on representative sample.

**Figure S2 | Increased *ALOX5AP* expression in MS tissue homogenates. (a-b)** mRNA expression of *ALOX5* (a) and *ALOX5AP* (b) in NNC (n=4) and MS tissue (n=7). Data is shown as box plots with median ± quartiles; whiskers extend to the minimum and maximum. For two groups, an unpaired t-test with Welch’s correction was used. Exact p-values are reported and statistical significance is set at *p <* 0.05 (red).

**Figure S3 | FLAP antagonism does not affect pro-inflammatory or pro-resolution gene expression profiles. (a-b)** Volcano plot visualizing differentially expressed genes in pro-inflammatory (LPS: 100 ng/ml, IFNγ: 20 ng/ml) (a) and pro-resolution (IL-4: 20 ng/ml, IL-13: 20 ng/ml) (b) hiPSC-derived microglia upon FLAP antagonism (fiboflapon, 0.5 µM). Data processing and normalization and subsequent differential expression (DE) analysis were executed using the edgeR (v4.0.16) and limma (v3.58.1) packages (27, 28). Differentially expressed genes (DEGs) were based on an adjusted p value of less than 0.05 and an absolute log2FC greater than 1, which were depicted in volcano plots produced using the ggven (v0.1.10) and EnhancedVolcano (v1.20.0) packages (30, 31).

**Figure S4 | FLAP antagonism does not affect COX-mediated LM production in hiPSC-derived microglia.** **(a)** COX product formation remains unaltered upon FLAPa (fiboflapon, 0.5 µM) treatment in resting, pro-inflammatory, and pro-resolution hiPSC-derived microglia. Data is shown as box plots with median ± quartiles; whiskers extend to the minimum and maximum. Data have been statistically tested for six groups by ordinary one-way ANOVA with Sidak’s correction.

**Figure S5 | Increased *ALOX5AP* expression in EAE spinal cord homogenates. (a-b)** mRNA expression of *Alox5* (a) and *Alox5AP* (b) in spinal cord tissue of experimental autoimmune encephalomyelitis (EAE) mice sacrificed at the chronic stage of the disease (29 days after EAE induction) (n=5) compared to healthy control mice (n=3). Data is shown as box plots with median ± quartiles; whiskers extend to the minimum and maximum. For two groups, an unpaired t-test with Welch’s correction was used. Exact p-values are reported and statistical significance is set at *p <* 0.05 (red).

**Figure S6 | Compound identification by High-Performance Liquid Chromatography-Mass Spectrometry. (a)** Mass spectra (recorded from HPLC eluents at 2 minutes) of brain samples from EAE mice treated with fiboflapon (2 mg/kg, i.p.), untreated and fiboflapon standard. Fiboflapon was identified as [C_38_H_43_N_3_O_4_S + H]^+^ peak (predicted m/z = 638.30527) that eluted at 2 minutes. M+1 and M+2 isotopologues are marked. **(b)** Retention time of the Fiboflapon was between 1.9 and 2.1 minutes, as was defined using a standard.

**Figure S7 | Prophylactic FLAP antagonism does not affect body weight loss or 5-LOX product formation in EAE. (a-b)** Body weight of EAE mice treated 9 days post-immunization with vehicle or FLAPa (fiboflapon 2 mg/kg, i.p. daily, prophylactic) and sacrificed at the chronic stage (n=13 per group) (a) or peak of the disease (n=6 per group) (b). **(c-d)** 5-LOX product ratios to arachidonic acid (AA) (c) and their absolute concentrations (d) in the spinal cord (SC) of vehicle- and FLAPa-treated EAE mice treated 9 days post-immunization with vehicle or FLAPa (fiboflapon 2 mg/kg, i.p. daily, prophylactic) and sacrificed at the chronic stage (n=13 per group). **(e-f)** 5-LOX product ratios to AA (e) and absolute 5-LOX product concentration (f) in the spinal cord (SC) of vehicle- and FLAPa-treated EAE mice treated 9 days post-immunization with vehicle or FLAPa (fiboflapon 2 mg/kg, i.p. daily, prophylactic) and sacrificed at the peak of the disease (n=6 per group). Data is shown as box plots with median ± quartiles; whiskers extend to the minimum and maximum. For two groups, a two-way ANOVA with Sidak’s correction was used. Exact p-values are reported and statistical significance is set at *p <* 0.05 (red).

**Figure S8 |** **Prophylactic FLAP antagonism does not alter immune cell infiltration or phenotype in chronic EAE. (a)** Representative immunohistochemical stainings for Ly6G/CD11b, CD3/CD45, MHCII/CD45 and F4/80/iNOS on SC tissue obtained from EAE animals treated with vehicle or FLAPa in the prophylactic setting and sacrificed at the chronic phase of the disease (n=13 per group); scale bar 200 µm. **(b-e)** Quantification of Ly6G^+^/CD11b^+^ cells (b, neutrophils), total CD45^+^ cells (c, immune cells), CD3^+^CD45^+^ cells (d, T cells), MHCII^+^CD45^+^ cells (e, activated macrophages/microglia), comparing vehicle-treated and FLAPa-treated EAE mice sacrificed in the chronic phase of the disease (n=6 per group). **(f-g)** Quantification of total F4/80^+^ cells (f) and percentage of iNOS^+^ cells within the F4/80^+^ population (g) comparing vehicle-treated and FLAPa-treated EAE mice at the chronic stage of the disease. **(h)** Quantitative PCR was used to determine mRNA levels of the pro-inflammatory genes *Ccl2*, *Ccl4*, *Ccl5*, *Il1b*, *Il6*, *Nos2*, and *Tnfa* to compare inflammation between treatment groups at the chronic stage of the disease. Data is shown as box plots with median ± quartiles; whiskers extend to the minimum and maximum. For two groups, an unpaired t-test with Welch’s correction was used (b-g) or the row factor was calculated using a two-way ANOVA with Sidak’s correction (h). Exact p-values are reported and statistical significance is set at *p <* 0.05 (red).

 **Figure S9 | Pharmacological FLAP antagonism has limited effects on immune cell infiltration.** **(a)** Disease scores of EAE mice treated 9 days post-immunization with vehicle or FLAPa (fiboflapon 2 mg/kg, i.p., daily, *n* = 10 per group) and sacrificed at the peak of disease (d17) for flow cytometric analysis of immune cell profiling. **(b)** Flow cytometric determination of leukocytes (CD45^+^) immune cell composition in blood, spleen, and spinal cord within the viable cell population. **(c-e)** Flow cytometric determination of immune cell composition in blood (c), spleen (d), and spinal cord (e) within the leukocyte population (CD45^+^). Data is shown as box plots with median + quartiles; whiskers extend to minimum and maximum. Data were statistically tested by two-way ANOVA with Sidak’s correction. Exact p-values are reported and statistical significance is set at p < 0.05 (red).

 **Figure S10 | Pharmacological FLAP antagonism skews immune cell subsets towards a subtle reduction in inflammatory phenotype.** **(a)** Inflammatory monocytes (Ly6C^high^) as percentage of total monocytes (F480^low^ CD11b^+^) in the blood and spleen (*n* = 10 per group). **(b)** Percentage of natural killer T cells (NKT cells, NK1.1^+^ CD3^+^) within the viable cell population of blood cells and splenocytes (*n* = 10 per group). **(c-d)** CD8^+^ and CD4^+^ T cell subsets in blood (c) and spleen (d) (*n* = 10 per group). **(e-h)** CD8^+^ (e) and CD4^+^ (f) T cell subsets and their respective naïve (CD44^low^ CD62L^high^), effector (CD44^high^ CD62L^low^), and memory (CD44^hi^ CD62L^hi^) subpopulations, B cell (CD19^+^) subpopulations (g), and dendritic cell (DC, CD11c^+^ MHCII^+^) subpopulations (h) in the blood (*n* = 10 per group). **(i-l)** CD8^+^ (i) and CD4^+^ (j) T cell subsets and their respective naïve, effector, and memory subpopulations, B cell subpopulations (k), and dendritic cell (DC) subpopulations (l) in the spleen (*n* = 10 per group). **(m-n)** B cell subpopulations (m), and dendritic cell (DC) subpopulations (n) in the spinal cord (*n* = 10 per group). Data is shown as box plots with median + quartiles; whiskers extend to minimum and maximum. Data were statistically tested by two-way ANOVA with Sidak’s correction. Exact p-values are reported and statistical significance is set at p < 0.05 (red).

**Figure S11 | Therapeutic FLAP antagonism does not affect body weight or downstream 5-LOX product formation in the EAE model. (a)** Body weight of EAE mice treated from the peak of the disease (17 days post-immunization) with vehicle or FLAPa (fiboflapon 2 mg/kg, i.p. daily, therapeutic) and sacrificed at the chronic stage (n=13 per group) **(b-c)** 5-LOX product ratio to arachidonic acid (AA) (b) or their absolute concentration (c) in the SC of vehicle- and FLAPa-treated EAE mice treated from the peak of the disease (17 days post-immunization) with vehicle or FLAPa (fiboflapon 2 mg/kg, i.p. daily, therapeutic) and sacrificed at the chronic stage (n=13 per group). Data have been statistically tested for two groups by a two-way ANOVA with Sidak’s correction**.**
